# Sunshine duration and solar radiation contributed to severe Bell’s palsy: An 11-year time series analysis based on a distributed lag non-linear model model

**DOI:** 10.1097/MD.0000000000034400

**Published:** 2023-07-21

**Authors:** Cuiyi Zhang, Fang Dong, Qi Wu, Jinlan Jin, Mengtao Li, Xiaojuan Xu, Zhihua Peng, Yuanting Chen, Meixia Ye, Xingli Liu, Lijun Wang, Yinqin Zhong

**Affiliations:** a Shenzhen Hospital of Guangzhou University of Chinese Medicine (Futian), Shenzhen, China.

**Keywords:** distributed lag non-linear model, meteorological factors, severe Bell’s palsy

## Abstract

Although previous studies have suggested that meteorological factors are associated with Bell’s palsy, articles on this topic are rare and the results are inconsistent. We aim to reveal the relationship between exposure to different meteorological factors and the onset of severe Bell’s palsy (SBP) with daily data. A case-crossover study based on time-series data was applied, and the minimum risk value of each climatic factor was set as the reference value. We fitted a distributed lag non-linear model (DLNM) which applied quasi-Poisson regression to evaluate the exposure-response association and the lag-response association of meteorological factors on the occurrence of SBP. The mode value and per-decile interval value of each meteorological factor were all included in the analysis. Sensitivity analyses were conducted to test the robustness of results. A total of 863 SBP patients (474 males and 389 females) from 7 hospitals in the Shenzhen Futian District were selected from January 2009 to February 2020. The highest relations effect was tested in the cumulative exposure-response result shown as follows; mean temperature at the minimum value 15.3°C with RR of 10.370 (1.557–69.077) over lag 0 to 13; relative humidity at the 30th value 71% with RR of 8.041 (1.016–63.616) over lag 0 to 14; wind speed at the 90th value 31 (0.1 m/s) with RR of 1.286 (1.038–1.593) over lag 0; mean air pressure at the 30th value 1001.4 (pa) with RR of 9.052 (1.039–78.858) over lag 0 to 5; visibility at the 80th value 26.5 (km) with RR of 1.961 (1.005–1.423) over lag 0 to 2; average total cloud cover at the max value 100 (%) with RR 1.787 (1.014–3.148) over lag 0 to 2; sunshine duration at the 10th value 0.1 (h) with RR of 4.772 (1.018–22.361); daily evaporation shows no relationship in the cumulative result; daily average solar radiation at the minimum value 0 (W/m^2^) with RR of 5.588 (1.184–26.382). There is a relationship between wind speed and the onset of SBP, while mean air pressure, visibility, and average total cloud cover, especially sunshine duration and solar radiation which showed a strong effect, may be associated with severe clinical symptoms of SBP. Mean temperature and relative humidity may affect the course of SBP.

## 1. Introduction

Bell’s palsy (BP), which was first described by Scottish anatomist, Sir Charles Bell, is an acute idiopathic peripheral facial nerve paralysis of sudden onset.^[1]^ It is the most common acute mono-neuropathy with typically self-limited, and the incidence of BP is approximately from 15 to 30 cases per 100,000 per year with an equal gender ratio. However, BP with severe symptoms may relate to a poor prognosis such as significant temporary oral incompetence, articulation difficulties, muscle contracture, eyelid closure disorder, and even more additional long-term injury.^[2]^ According to relevant statistics there are as many as 30% of patients who do not recover completely, thus it may cause a huge impact on patient’s appearance, quality of life, and psychological wellbeing.^[3]^

It has been reported that the development of BP may be caused by the inflammation or compression of seventh cranial nerve; however, the specific triggers still remain unclear. Recently, the effect of climate on diseases has received a lot of attention. Some researchers suggested that meteorological factors such as temperature have an indirect impact on BP that lower temperatures were associated with BP’s high incidence; however, the conclusions are inconsistent.^[4–6]^ What’s more, Young Kim et al reported that the high concentrations of air pollutant NO_2_ may directly influence the development of BP by oxidative stress and inflammatory response.^[7]^ These studies suggest that climatic or meteorological factors may be associated with BP.

The atmospheric system is composed of a variety of meteorological factors, each of which has complex interactions, such as non-liner relationship, cyclicality and predictability. Therefore, simplistic, linear, and cross-sectional studies may lead to biased research results. On the other hand, there were few studies focused on the relationship between climate factors and sever Bell’s palsy (SBP), especially for the progress of SBP research. Thus, our aim was to reveal the comprehensive association between climatic factors and SBP. Firstly, we collected all existing observable meteorological factors to evaluate the overall effect of weather condition on the occurrence of SBP. What’s more, we applied a distributed lag non-linear model (DLNM), which is based on cross-basis calculations, to calculate the time effect and lag effect of meteorological factors on the occurrence of SBP. Last but not the least, all the analysis of SBP patients used data at the time of onset rather than the time of visit.

## 2. Material and methods

### 2.1. Study area

**Figure 1.**
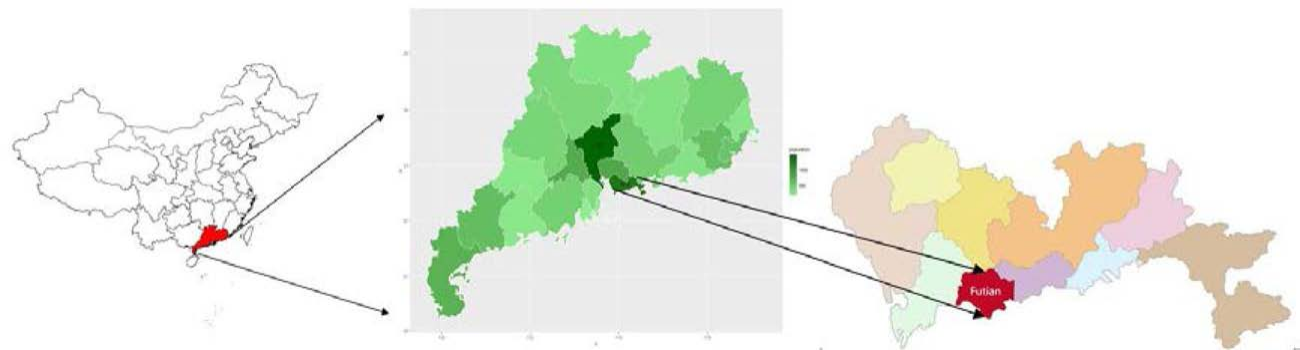
The geographical location of Shenzhen and Futian district.

Shenzhen is a coastal city in southern China, located between 113°46′ to 114°37′ east longitudes and 22°24′ to 22°52′ north latitudes, which area is long and narrow from east to west. It has a long summer and short winter with humid air and a subtropical monsoon climate. Futian district is located in the middle part of Shenzhen; she is also the political center of Shenzhen which has a total area of 78.8 square kilometers and a permanent population of 1633700. Figure 1 shows the geographical location of Futian district of Shenzhen, China.

### 2.2. Case selection

In view of the fact that most BP patients show recovery without intervention within 2 weeks after onset of symptoms, we only selected inpatients with the severe conditions based on admission assessment, such as severe manifestations, longer duration of the disease, may have a poor prognosis, and may involve complex treatments. For this case-crossover study, time-series data from 7 hospitals in Shenzhen Futian District Medical and Health Group (The Eighth Affiliated Hospital of Sun Yat-sen University [Shenzhen Futian], Shenzhen Hospital of Guangzhou University of Chinese Medicine [Futian], Futian District Maternal and Child Health Hospital, The Second People’s Hospital of Futian District, Futian District Chronic Disease Prevention and Treatment Hospital, Futian District Rheumatism Specialist Hospital, Shenzhen Anorectal Hospital of Traditional Chinese Medicine [Futian]) were included in the study. We obtained daily data on the number of severe Bell’s palsy patients who were admitted to hospital from January 2009 to February 2020 (based on the 10th international Classification of Disease ICD-10 code G51.001). All cases were then carefully selected by 2 Specialists by viewing medical records with the following inclusions;

1. Unilateral facial nerve paresis or paralysis without identifiable cause;
2. Bell’s palsy patients whose assessment are level 4 or higher (Grade ≥ 4) based on House-Brackmann facial nerve grading scale were definite as severe Bell’s palsy;
3. Medical records are complete and can be accurately traced back to the time of onset;
4. Permanent residence is Shenzhen and 7 days before onset to onset day all stay in Shenzhen;
5. Only the first record of multiple admissions.

Exclusion criteria

1. Differential diagnosis with the following diseases: Central facial paralysis; facial paralysis left by surgery and traumatic facial nerve damage; facial paralysis caused by posterior fossa lesions such as acoustic neuroma, skull base meningitis, multiple sclerosis, etc.; facial paralysis caused by tumor factors; Guillain-Barré syndrome Neural Lyme disease; acute purulent otitis media, chronic otitis media, mastoiditis, external otitis, etc. caused by otogenic facial paralysis; congenital facial nerve palsy; pregnant women;
2. The date of patient onset is uncertain
3. Repeat admission patients.

### 2.3. Meteorological data

There are 11 weather monitoring stations in Shenzhen to detect weather conditions. Considering that the meteorological data of different districts will differ by 1 to 2 units, we only used data from the weather station that located in Futian to accurately assess the climate effects on Bell’s palsy.

A total of 10 meteorological factors were analyzed in our research which mean temperature (°C), Relative Humidity (%), wind speed (0.1 m/s), average water pressure (hpa), mean air pressure (hpa), visibility (km), average total cloud cover (%), and daily average solar radiation (W/m^2^) using the daily average value of 4 times at 2 am, 8 am, 2 pm, and 8 pm. Meanwhile, the sunshine duration (h) and daily evaporation (mm) was using the cumulative value of the day. All data provided by Shenzhen Meteorological Service Center. Each meteorological factor has a time series chart before being included in the model analysis; details see in Figure S1, Supplemental Digital Content, http://links.lww.com/MD/J331 which illustrates the pictures.

### 2.4. Statistical analysis

Firstly, we applied a standard time-series quasi-Poisson regression which could control the over-dispersion, to define the relationship of weather changes and Bell’s palsy’s occurrence.

Then, a DLNM was built to evaluate the conventional exposure-response association and the additional lag-response association of meteorological factors on the occurrence of severe Bell’s palsy, respectively. The DLNM model is based on build basis and cross-basis matrices that used to describe simultaneously non-liner and delayed effects between weather predictors and the disease onset, and built as follows.

log [E(Y_t_)] = α + Σ cb(W_t_, maxlag) + ns(time, 7*12) + dow_t`_

In the model, t is the date of observation; Y_t_ is the observed daily onset number of severe Bell’s palsy; α is the intercept; cb means cross-basis matrix obtained by applying DLNM; W instead of included weather factors; maxlag was the setting max lag days; ns represent the natural spline function; time with 7 degrees of freedom per year is an indicator of day of the week to control for seasonal and long-term trends. The number of knots is set to df-1-intercept for “ns,” and df-degree-intercept for “bs.” dow is a dummy variable for the day of the week. The df which modeled by a quadratic B-spline was set at 3 for the exposure-response relationship calculation, and the degree of freedom of the lag-response relationship was set at 5 with a natural cubic B-spline.

Most patients with BP showed some recovery without intervention within 2 weeks after onset of symptoms.^[2]^ Therefore, we set the maximum lag as 14 days to capture the complete lag-response curve to avoid overestimating the effect of the climatic factors. What’s more, both the single lag impact and the cumulative effects of meteorological factors on the occurrence of SBP were calculated. The minimum risk value of each climatic factor by derived from the BLUP (best linear unbiased prediction) of the overall cumulative exposure-response association were set as the centering value of the quadratic B-spline. The relative risk of both the mode value and the deciles spacing value in 10 years were analyzed, which may represent the relationship between common climate conditions in daily life and the prevalence of SBP in Shenzhen.

### 2.5. Sensitivity analysis

We did all analyses with R (version 4.0.0), using the packages *dlnm* and *splines*. all tests were two-sided, with *P* values < .05 as statistical significant. A sensitivity analysis was also performed to test the robustness of the model calculation results that changing the df = 5/6 in ns function and time.

### 2.6. Ethical review

This study was approved by the Ethics Committee of the Shenzhen Hospital of Guangzhou University of Chinese Medicine (Futian) (no. GZYLL(KY)-2020-030).

## 3. Results

**Figure 2.**
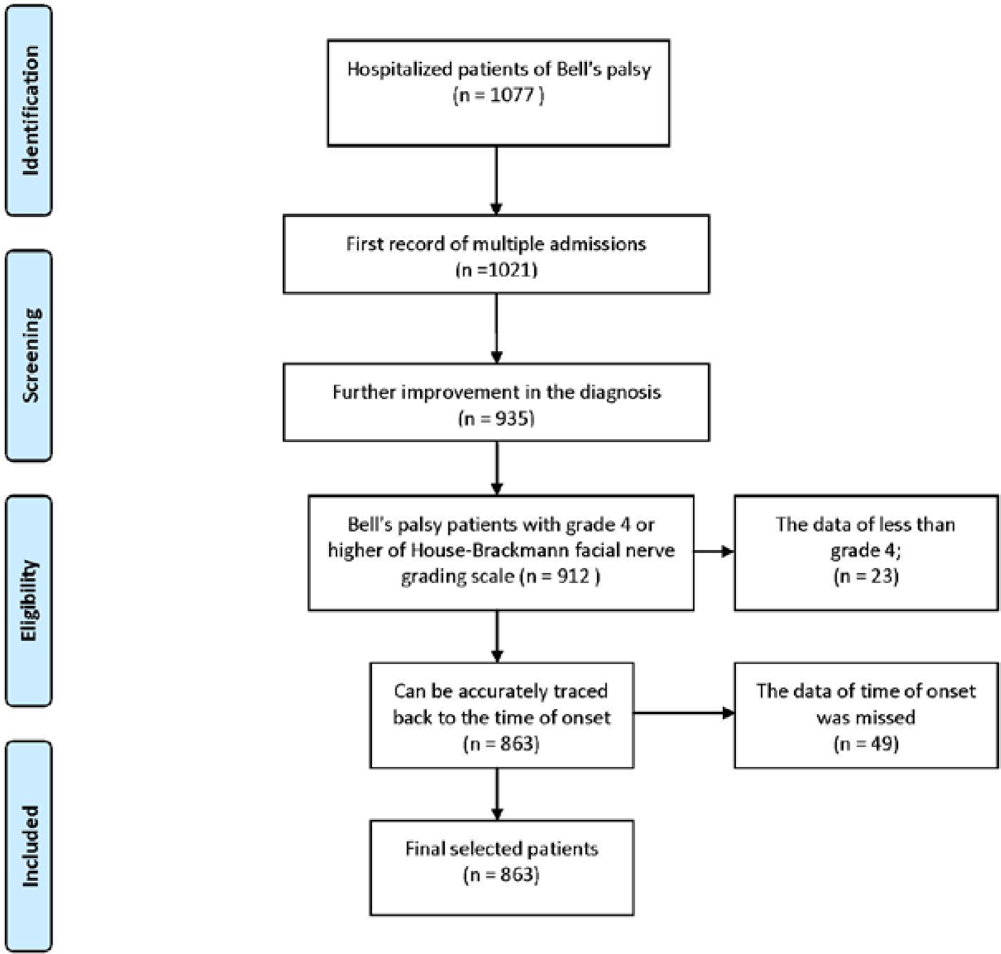
The case selection process of severe Bell’s palsy patients.

**Figure 3.**
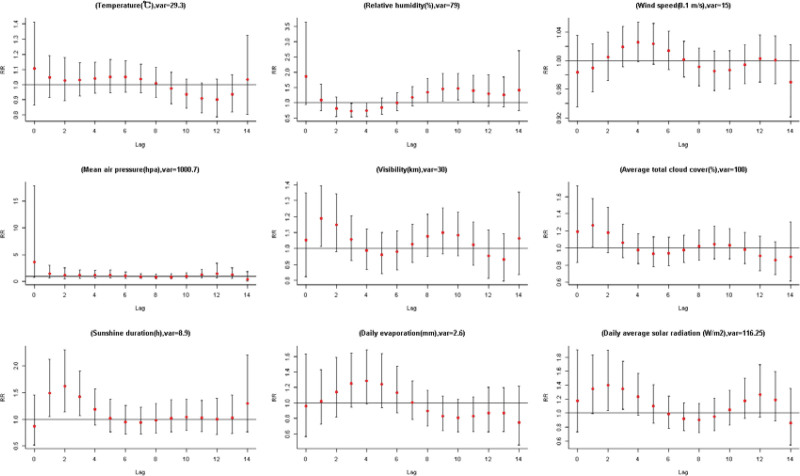
The single day lag-response curves on SBP for the mode value of 9 weather factors at different lags in Shenzhen Futian district, 2009–2020. SBP = severe Bell’s palsy.

There were a total of 863 severe Bell’s palsy patients finally selected from all the people diagnosed with SBP; the selection process was demonstrated in Figure 2. Table 1 shows the descriptive statistics information of all patients. The general information for meteorological factors in Shenzhen from 2009 to 2020 are shown in Table 2. The result of the mode value of weather factors to the disease which represents the most common situation in 10 years are shown in Figure 3. In order to facilitate the understanding of the results, we artificially defined the lag days ≤ 7 days as the short-term effect, when 7 days < lag days ≤ 14 days it named as the relative long-term effect.

**Table 1:** Descriptive statistics for Bell’s Palsy patients in Shenzhen Futian district, 2009–2020.

Variables	All	Left	Right
Number	863	422	441
Male	474	244	230
Female	389	178	211
Age	41.87 ± 13.68	42.28 ± 13.58	41.47 ± 13.77
≤45	32.80 ± 6.84	33.22 ± 6.67	32.40 ± 6.98
>45	55.82 ± 9.02	56.12 ± 9.01	55.53 ± 9.05
Average hospital stay	11.75 ± 4.72	11.65 ± 4.84	11.84 ± 4.61
Diabetes	98	39	59
Hypertension	159	72	87
Hyperlipidemia	114	51	63
Hyperuricemia	79	37	42

**Table 2:** Descriptive statistics for 9 daily meteorological factors in Shenzhen Futian district, 2009–2020.

Variables	Mean ± SD	Min	P_25_	P_50_	P_75_	Max	M_0_
Mean temperature (°C)	23.25 ± 5.50	3.5	19.2	24.5	28	33	29.3
Relative humidity (%)	74.71 ± 12.99	19	68	77	84	100	79
Wind speed (0.1 m/s)	20.61 ± 8.04	3	15	19	25	67	18
Mean air pressure (hpa)	1005.42 ± 6.43	983.1	1000.6	1005.4	1010.3	1027.3	1000.7
Visibility (km)	18.09 ± 7.82	1.8	11.3	17.8	25.0	39.3	30
Average total cloud cover (%)	64.59 ± 25.05	0	49.0	70.0	83.0	100.0	100
Sunshine duration (h)	5.23 ± 3.78	0	1.4	5.6	8.7	12.5	0
Daily evaporation (mm)	3.30 ± 1.62	0	2.1	3.2	4.3	12.0	2.6
Daily average solar radiation (W/m^2^)	144.45 ± 69.18	15	91.8	137.8	189.8	335	116.3

M_0_ = mode.

### 3.1. The associations between meteorological factors and SBP over their lag effects

#### 3.1.1. The short-term effect (lag day ≤ 7 days).

There is a significant association between sunshine duration and SBP onset which shows a strong correlation over lag 1, lag 2, and lag 3 days, although characterized by a high RR. However, there are no relationships of mean temperature and mean air pressure on SBP in short-term effect. Daily average solar radiation shows a moderate effect on SBP onset over lag 1, lag 2, and lag 3 at some decile value. And there is also a medium impact of relative humidity on SBP over lag 3 and lag 4 which is similar to daily evaporation. Other slight relationships of visibility and average total cloud cover on SBP onset over lag 1 to lag 3 at different decile value are shown in Table S1, Supplemental Digital Content, http://links.lww.com/MD/J333 which illustrates lag-specific relative risks of 9 meteorological factors. Details about Lag-response curve for lag 0 to lag 14 of 9 meteorological factors in Shenzhen Futian district from 2009 to 2020 are shown in Figures S2 to S16, Supplemental Digital Content, http://links.lww.com/MD/J332 which illustrates short-term effect of 9 meteorological factors.

#### 3.1.2. The relative long-term effect (7 days < lag day ≤ 14 days).

Relative humidity shows closely related to SBP that there is a strong significant association between the minimum value and SBP over lag 8, lag 9, and lag 10. Some other Relative humidity deciles also have a moderate long-term effect on SBP over lag 8 to lag 11. Mean temperature shows a slight impact on SBP over lag 10, lag 11, and lag 13. Visibility also shows a slight association to SBP at 10.1 and 12.5 km over lag 8, lag 9, and lag 10. However, there were no long-term effects of other weather factors on SBP has been proved; for details, see Table S1, Supplemental Digital Content, http://links.lww.com/MD/J333.

### 3.2. The cumulative associations between meteorological factors and SBP over their cumulative lag effects

#### 3.2.1. The cumulative short-term effect (lag ≤ 7 days).

There is a strong cumulative correlation of daily average solar radiation on SBP, especially over lag 0 to 3, lag 0 to 4, and lag 0 to 5 days at each decile value. Compare to the max solar radiation of 335 W/m^2^, the value of 15 W/m^2^ is relative risk to SBP that RR is 2.423 with its 95% CI (1.052–5.581) over lag 0 to 1, and the RR is 3.400 with its 95% CI (1.207–9.581) over lag 0 to 2. The sunshine duration shows a significant association to the SBP, especially over 0 to 4 days at each decile value. There is also a great cumulative effect of mean air pressure on SBP about the 10th and 20th percentiles over lag 0 to 2 to lag 0 to 5, that the 30th and 40th percentiles over lag 0 to 4 to lag 0 to 5, respectively. The slight cumulative relationship of weather factors to SBP shown in visibility and average total cloud cover, details see in Table S2, Supplemental Digital Content, http://links.lww.com/MD/J334 which illustrates cumulative effect of 9 meteorological factors. However, there are no effects of mean temperature, relative humidity; wind speed and daily evaporation on severe Bell’s palsy onset have been proved.

#### 3.2.2. The cumulative long-term effect (7 days < lag ≤ 14 days).

**Figure 4.**
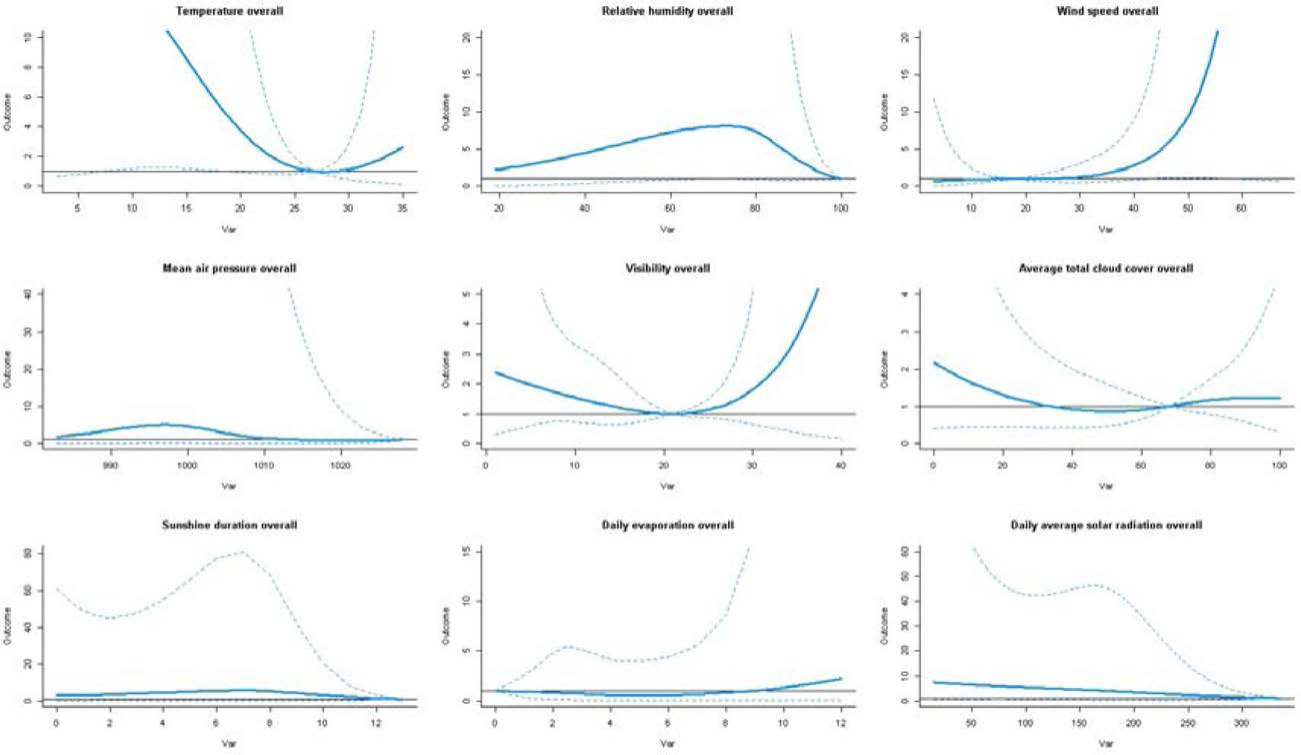
The overall effect of each meteorological factor on SBP at lag 0–14 in Shenzhen Futian district, 2009–2020. SBP = severe Bell’s palsy.

**Figure 5.**
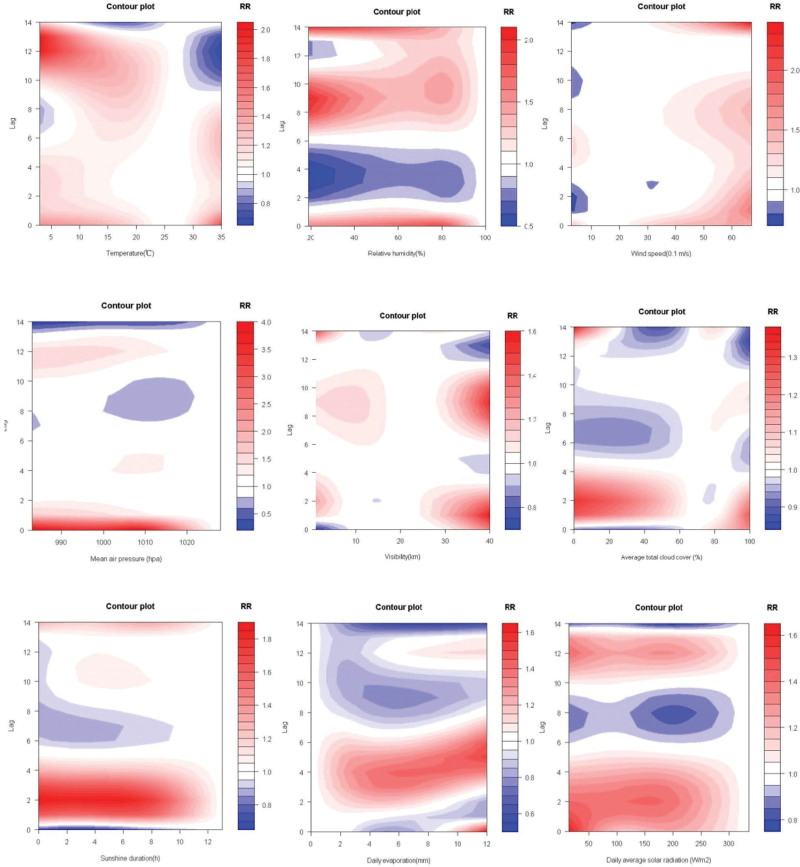
The countour graphes of comprehensive summary of exposure-lag-response association between 9 climatic factors and SBP. SBP = severe Bell’s palsy.

**Figure 6.**
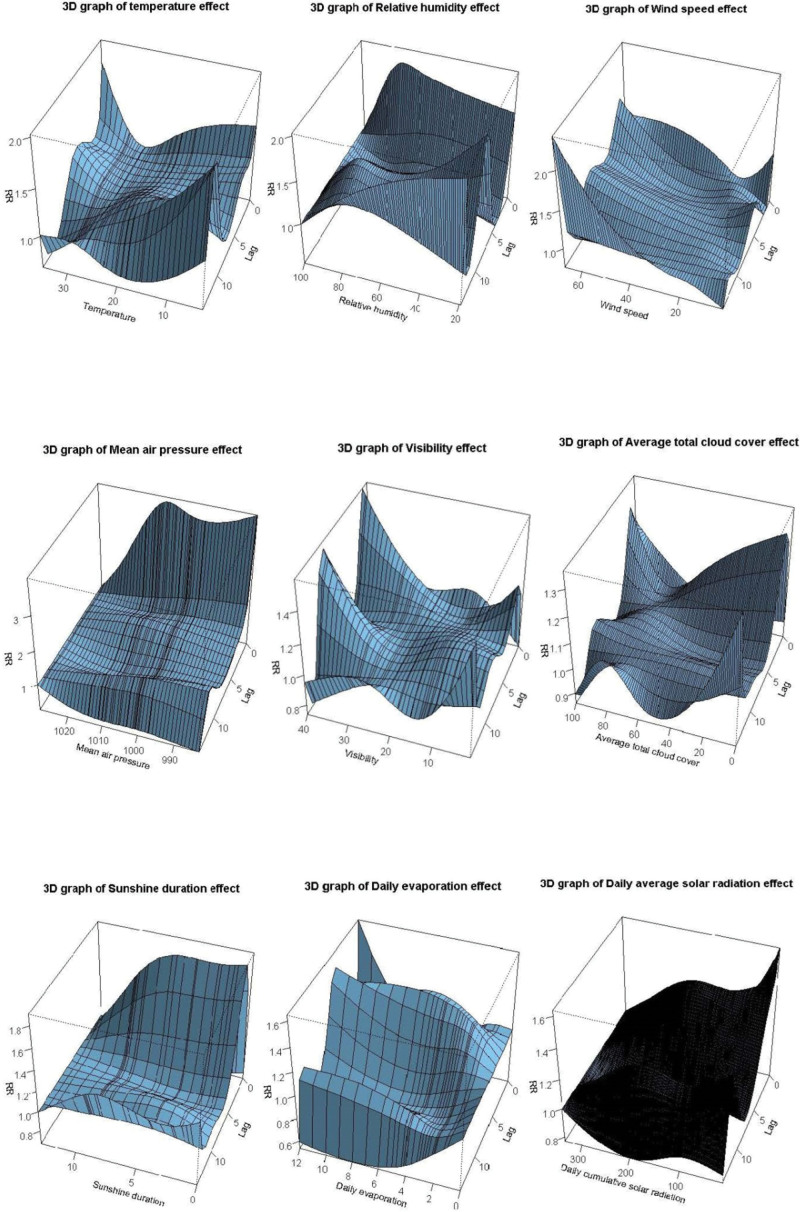
The 3-D plots of comprehensive summary of exposure-lag-response association between 9 climatic factors and SBP. SBP = severe Bell’s palsy.

Only mean temperature and relative humidity demonstrate a significant cumulative long-term effect on SBP. The cumulative effect of temperature was only calculated after 11 days that at 10th, 20th, 30th, and 40th percentiles over lag 0 to 11 days to lag 0 to 14 days. Compare to the max relative humidity 100%, the value of 71% is risk to SBP with RR 8.041 and 95% CI (1.016–63.616) over lag 0 to 14. The overall effect plots are shown in Figure 4; contour graphs and 3-D plots of comprehensive summary of the three-dimensional exposure-lag-response association are shown in Figures 5 and 6; for details, see Table S2, Supplemental Digital Content, http://links.lww.com/MD/J334.

### 3.3. Sensitive analyses

When we changed the value of time df from 5/6/7 in ns function, the RR result of mode value for 9 climatic factors over lag 0 to lag 14 days change slightly, proving that our result is robust; see Table S3, Supplemental Digital Content, http://links.lww.com/MD/J335.

## 4. Discussion

Our study has revealed the association between meteorological factors and SBP of both time effect and lag effect, which included 863 patients from 7 hospitals over 11 years. It has indicated that the relative low temperature (compare to 27°C) showed a high correlation with SBP at 11 days after the onset. When the relative humidity is 71% (compare to 100%), the relative risk of SBP will increase by 704% (RR = 8.041, 95% CI: 1.016–63.616) over lag 0 to 14 days. When the wind speed is at 2.6 and 3.1 m/s, the risk of SBP will increase by 18.1% (RR = 1.181, 95% CI: 1.008–1.384) and 28.6% (RR = 1.286, 95% CI: 1.038–1.593), respectively. At the same time, we newly found that the sunshine duration (compare to 13 hours) and daily average solar radiation (compare to 335 w/m^2^) made great contributions to SBP at 3 to 4 days after the onset and 3 to 5 days after the onset, respectively. In addition, the analysis of the results under the most common climatic conditions in Shenzhen shows that there are 4 climate factors related to SBP, among which the visibility and average total cloud cover contribute to SBP over lag 1; while the sunshine duration contributes to SBP over lag 1, lag 2, and lag 3; and the daily average solar radiation contribute to SBP over lag 2 and lag 3.

To our knowledge, this is the only study to date that reveals the association between SBP and climatic conditions in a Chinese population. In order to accurately clarify the effect of meteorological factors on SBP, we asked each case to provide its onset date and excluded patients who were repeatedly admitted to the hospital. What’s more, the model was calculated based on the daily personal SBP data and predicted the 14-day lagging effect of each meteorological factor at each decile spacing value. The periodic impact of seasonal factors on the incidence of SBP and its non-linear correlation were also considered in the modeling process. Thus, we built a DLNM model to evaluate the exposure-lag-response effects between the climatic factors and SBP onset to summarize overall-cumulative exposure-response and lag-specific exposure-response.

BP is a common cranial neuropathy causing acute unilateral lower motor neuron facial paralysis, which symptoms generally resolve within weeks to months.^[8]^ However it has been reported that there are as many as 30% patients showing delayed or incomplete recovery,^[9]^ and approximately 25% of patients with BP suffering severe sequelaes such as persist facial weakness, contracture, or hemifacial spasm.^[10]^ Thus, the long-term incomplete recovery of the facial nerve imposes a great psychological burden on patients and seriously affects their quality of life. The exact pathogenesis of BP is still unclear, while viral infection, autoimmune inflammatory disease, microangiopathic ischemia, and inflammatory neuritis are all thought as potential contributors to the development of BP.^[11]^ A viral infection is the most acceptable one of the possible causes, which has been detected in 31% to 79% of cases, especially herpes simplex virus (HSV).^[12,13]^ One possible cause has been suggested that after a primary infection these latent viruses reside in the centered around geniculate ganglion and reactivation induced by immune modulation which caused local damage to the myelin.^[12,14,15]^ Although there is controversy about this conclusion,^[16]^ antiviral treatment is recommended by the clinical guidelines.^[2]^

Previous studies have revealed that meteorological factors are associated with the incidence of BP. It has been reported that there is a statistically significant monthly and seasonal variation of admitted cases of BP which occur most in cold season,^[4,17–19]^ and strong wind speeds are considered to related with BP occurrence.^[5]^ The possible mechanism of environmental factors causing disease is the hypothesis that specific climatic conditions may facilitate HSV-1 infection that renders human tissues more vulnerable to HSV-1 reactivation.^[4]^ What’s more, seasonal climatic changes may stimulate vascular-ischemic mechanism or infection-inflammatory mechanism to lead to BP.^[20]^ Therefore, climatic factors may play an important role in the pathogenesis of the disease.

It has been suggested that ultraviolet radiation (UVR) can affect both cellular immune response and humoral immunity, which impair the resistance to infections of different systems by inhibiting the systemic and local immune responses to multiple antigens. According to El-Ghorr’s research, UVR exposure given prior to secondary infection with HSV may reduce the Major Histocompatibility Complex (MHC) class II expression of dendritic cells that lead to modulation of local antigen presentation in mice model.^[21]^ In human research, UVR may affect humoral immunity by suppressive intrinsic activity of B cells.^[22]^ In addition, some articles reported that UVR may cause HSV recurrence in some people and aggravated symptoms of neurological infection.^[23–25]^ The quantified relationship between the viral load, UVR exposure and clinical symptom was proved by some research.^[26]^ It was estimated that with prolonged exposure at noon on a clear sky, neurological symptoms will increase in HSV infection humans.^[25]^

In our research, the single day lag-response shows that daily average solar radiation is related with SBP onset over lag 1 to 3. In the cumulative effect results, daily average solar radiation has shown a strong contribution to SBP at some specific value over lag 3 to 6 days; its cumulative effect reaches its peak on the fifth day. However, with the increase amount of radiation, the correlation does not show a clear consistent relationship. The possible reason for this phenomenon may be due to the complex component of solar radiation. when the amount of radiation reaches a certain level, the heat also shows effects to the diseases and interacts with ultraviolet rays. The sunshine duration may also affect the occurrence and clinical manifestation of SBP. The specific lag day result shows that there is an association between sunshine duration and SBP onset over lag 1 to 3 at each decile value. A strong cumulative correlation between sunshine duration and SBP was calculated over lag 3 to 4. We may hypothesize that the daily average solar radiation and sunshine duration may related to the severity of the symptoms after the onset of SBP. At the same time, based on the analysis of the most common climate conditions, we found that only average solar radiation and sunshine duration contributed to SBP, thus our results provide a new prevention strategy that sunshine protection or reducing the length of exposure to the sunshine could prevent the occurrence of facial paralysis, especially in subtropical monsoon climate areas.

Some studies have revealed that exposure to cold temperature may be closely related to BP, although the conclusions are inconsistent. For example, it has been reported that low temperature or sharp changes in temperature might cause the facial vasoconstriction reflex, triggering the vascular-ischemic mechanism.^[27]^ In addition, it is suggested that prolonged cold could enhance reactivation of the virus in ganglionic cells.^[4,28]^ In our study, daily mean temperature shows long-term cumulative exposure-response to SBP over lag 11 to lag 14 at lower value which is partially similar to the conclusions of other studies that cold weather is associated with BP.^[4,18,19]^ However, it is worth noting that the correlation between temperature and the occurrence of SBP only became apparent after 10 days, which suggested that temperature may be related to the prolongation of disease course.

We also found that the long-term cumulative effect to SBP was also shown in relative humidity over lag 14 days at 30th (RR = 8.041, 95% CI: 1.016–63.616). Thus, humidity may play an important role in the progression of BP. There is an immediate effect of wind on SBP that a higher wind speed may cause the disease occurrences but with a slight contribution. Although a study from Korea has reported that maximum wind speed 1 day before the onset of BP is statistically higher than that of no onset BP days,^[5]^ our results show a lag 0 effect. Franzke et al^[6]^ has reported that there was no significant association between meteorological parameters (atmospheric pressure, relative air humidity and temperature) and the risk of BP at any lag time (max lag = 3) in the overall population. However, in our research relative air humidity shows a moderate impact on the onset of SBP over lag 3 at minimum value, 50th, 60th, 70th, 80th, and 90th. This difference in results might be due to the different number of factors included in the model analysis and different model calculation methods. And some potential undiscovered factors may cause bias in results. Visibility was firstly discovered shown a slight effect on SBP at 26.5 km over lag 0 to 2 (RR = 1.196, 95% CI: 1.005–1.423) and lag 0 to 3 (RR = 1.245, 95% CI: 1.018–1.522). Average total cloud cover was also shown a newly effect on SBP at 100% over lag 0 to 2 (RR = 1.787, 95% CI: 1.014–3.148). A case-control study from South Korea^[7]^ showed that the concentration of NO_2_ for 60 days might be a risk factor to BP, however, other meteorological factors which including temperature, humidity, atmospheric pressure, SO_2_, and CO were not associated with BP. J. I. de Diego’s study^[26]^ demonstrated that there was no link between air pollutants (SO_2_, CO, O_3_, NO_2_, NO, and CH_4_) and the number of BP. Thus, the relationships of pollutants and the incidence of BP are still controversial.

As stated above, our limitation was failing to explore the relationship between pollutants and the pathogenesis of BP by introducing multiple pollutants into the model. Second, we only analyzed data on patients and weather in Futian District, the data from other regions in Shenzhen were not included, which made it difficult to generalize the results to the whole population. Third, our study was specially focused on the number of inpatients, individual vulnerability factors, such as age and genders did not do subgroup analysis. Finally, there was some misclassification bias which came from ecological research. Therefore, our conclusions still need to be used with caution, and studies with larger sample sizes need to be proven in the future.

We could conclude that wind speed is related to the onset of SBP, while mean air pressure, visibility, and average total cloud cover, especially sunshine duration and solar radiation which showed a strong effect, are associated with the severity of clinical symptoms of SBP. Mean temperature and relative humidity might affect the course of SBP.

## Acknowledgments

We appreciated the daily climatic data support by Shenzhen Meteorological Bureau.

## Author contributions

**Conceptualization:** Xingli Liu, Lijun Wang, Yinqin Zhong.

**Data curation:** Mengtao Li.

**Formal analysis:** Mengtao Li.

**Investigation:** Qi Wu.

**Methodology:** Jinlan Jin.

**Software:** Xiaojuan Xu, Yuanting Chen.

**Supervision:** Qi Wu, Xingli Liu.

**Validation:** Zhihua Peng, Xingli Liu, Lijun Wang.

**Visualization:** Xiaojuan Xu, Meixia Ye, Lijun Wang.

**Writing – original draft:** Cuiyi Zhang, Fang Dong.

**Writing – review & editing:** Fang Dong.

## Supplementary Material
