# Supplementary figures and images for: Sunshine duration and solar radiation contributed to severe Bell’s palsy: An 11-year time series analysis based on a distributed lag non-linear model model
Source: Medicine (Baltimore). 2023 Jul 21;102(29):e34400. doi: 10.1097/MD.0000000000034400 (PMC10662859; doi:10.1097/MD.0000000000034400)

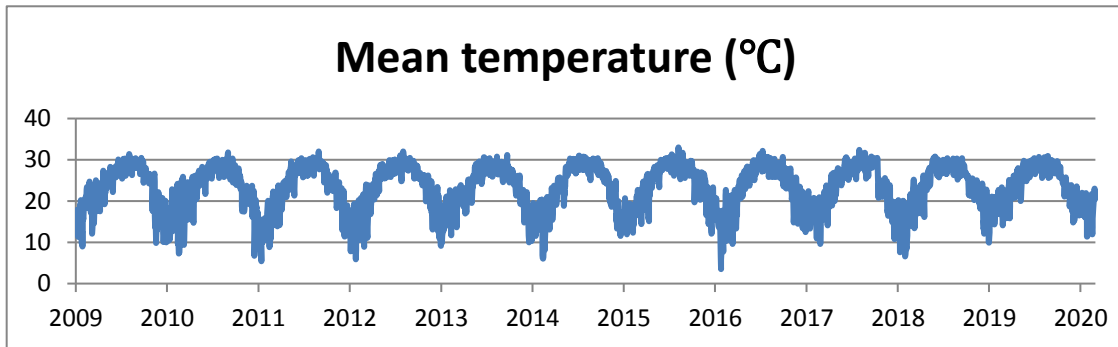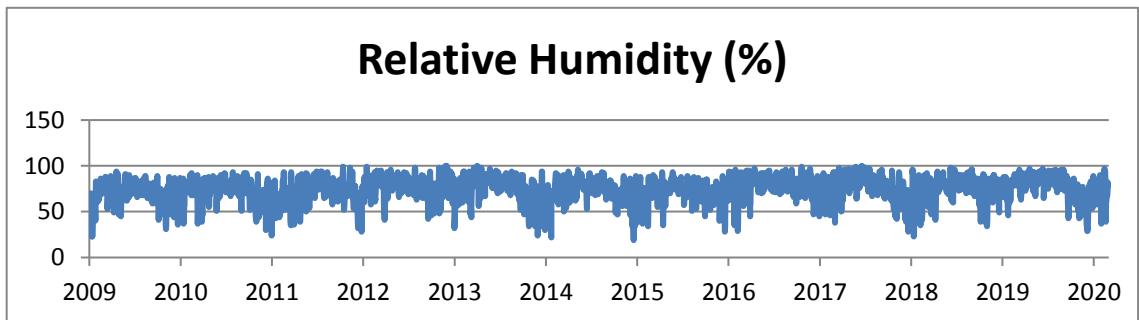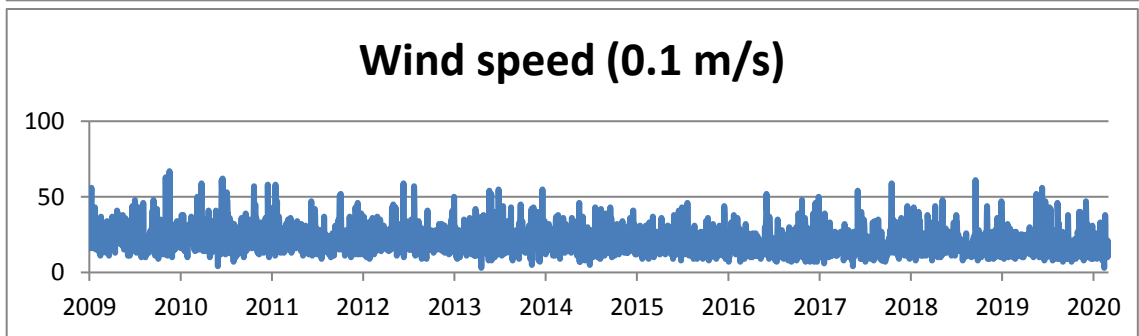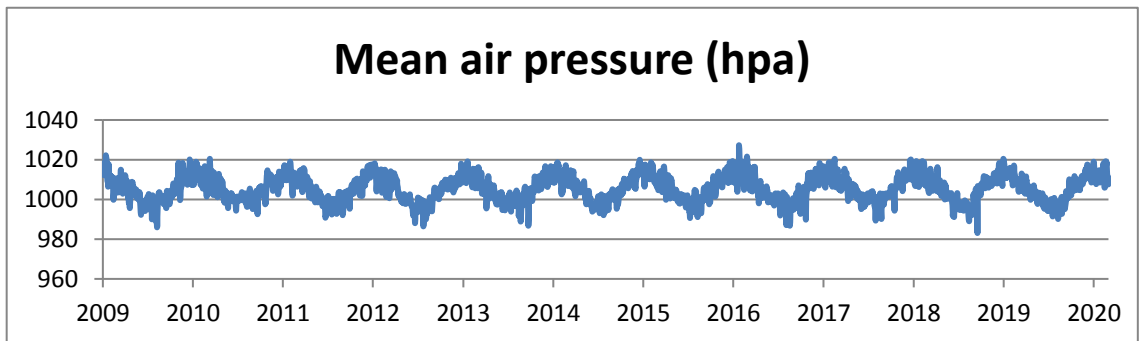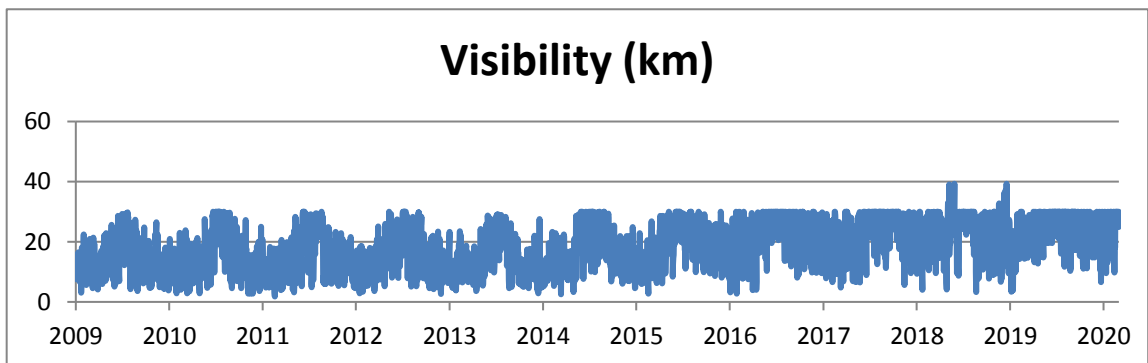

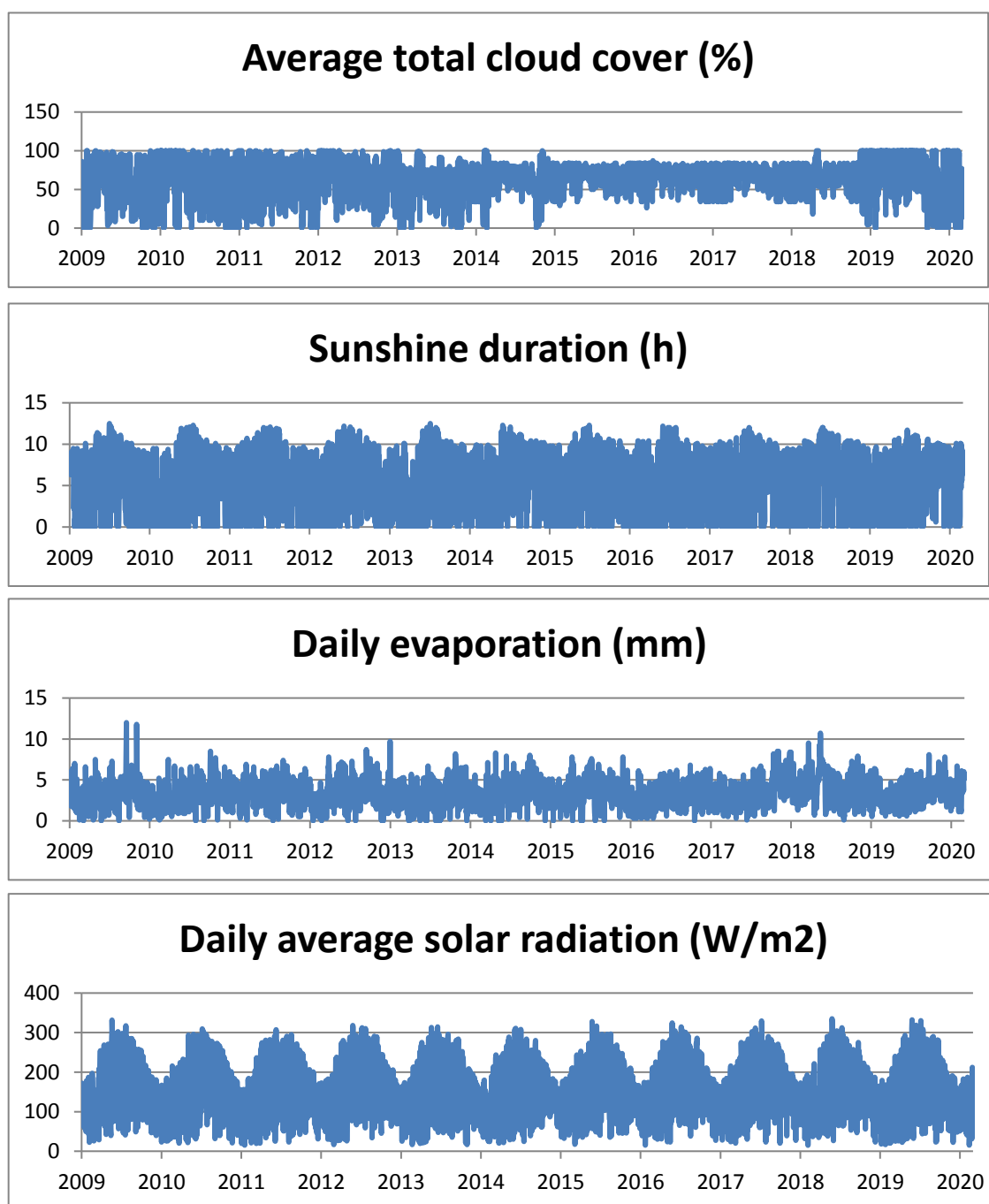

**Fig.S1** Time series diagram of 9 meteorological factors in Shenzhen Futian district, 2009-2020

Supplement: Supplementary file 1 [file medi-102-e34400-s001.pdf]
