# Supplementary material for: Sunshine duration and solar radiation contributed to severe Bell’s palsy: An 11-year time series analysis based on a distributed lag non-linear model model
Source: Medicine (Baltimore). 2023 Jul 21;102(29):e34400. doi: 10.1097/MD.0000000000034400 (PMC10662859; doi:10.1097/MD.0000000000034400)
Supplement: Supplementary file 3 [file medi-102-e34400-s003.pdf]

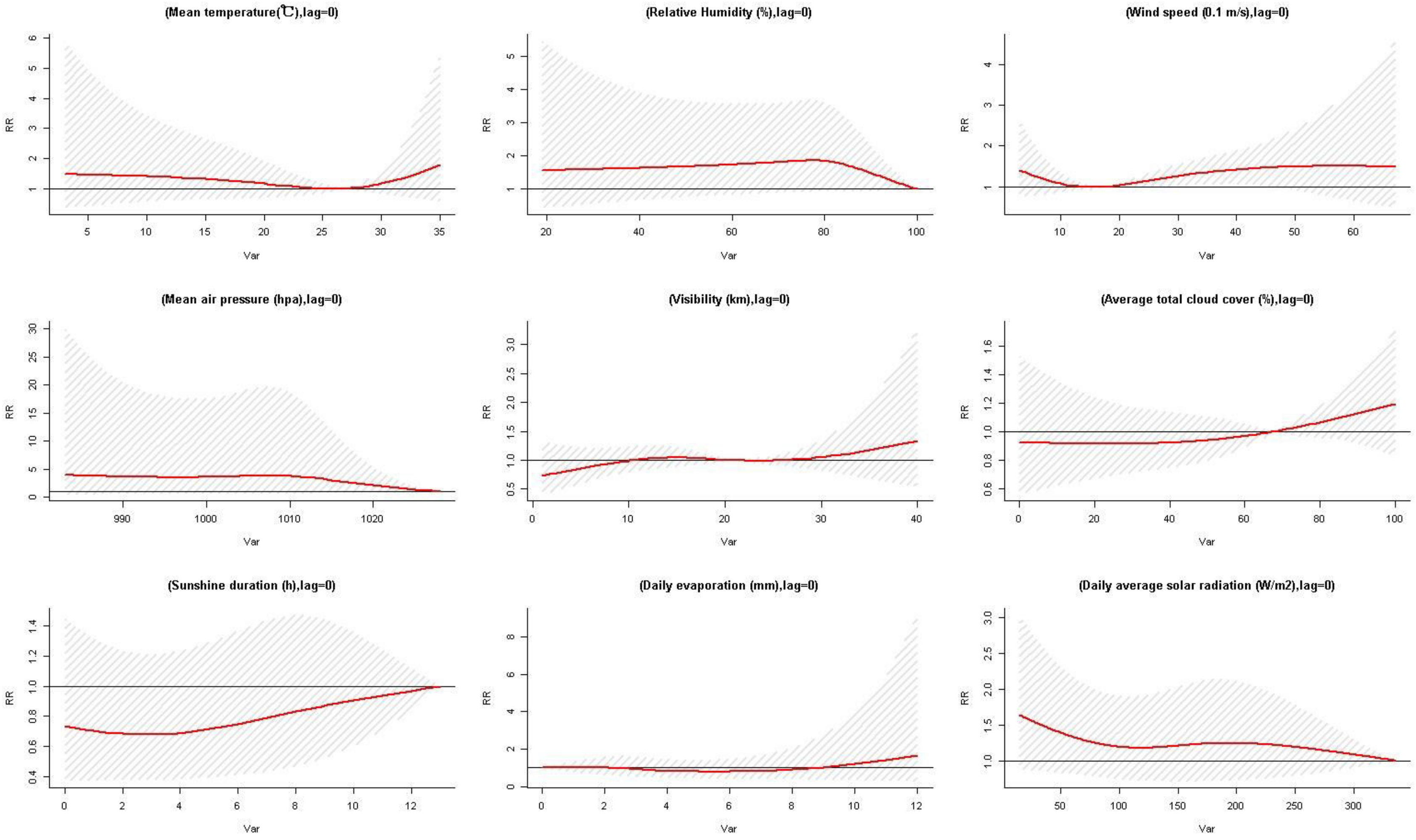

Fig.S2 Lag-response curve for lag0 effect of 9 meteorological factors on severe Bell's palsy in Shenzhen Futian district, 2009-2020

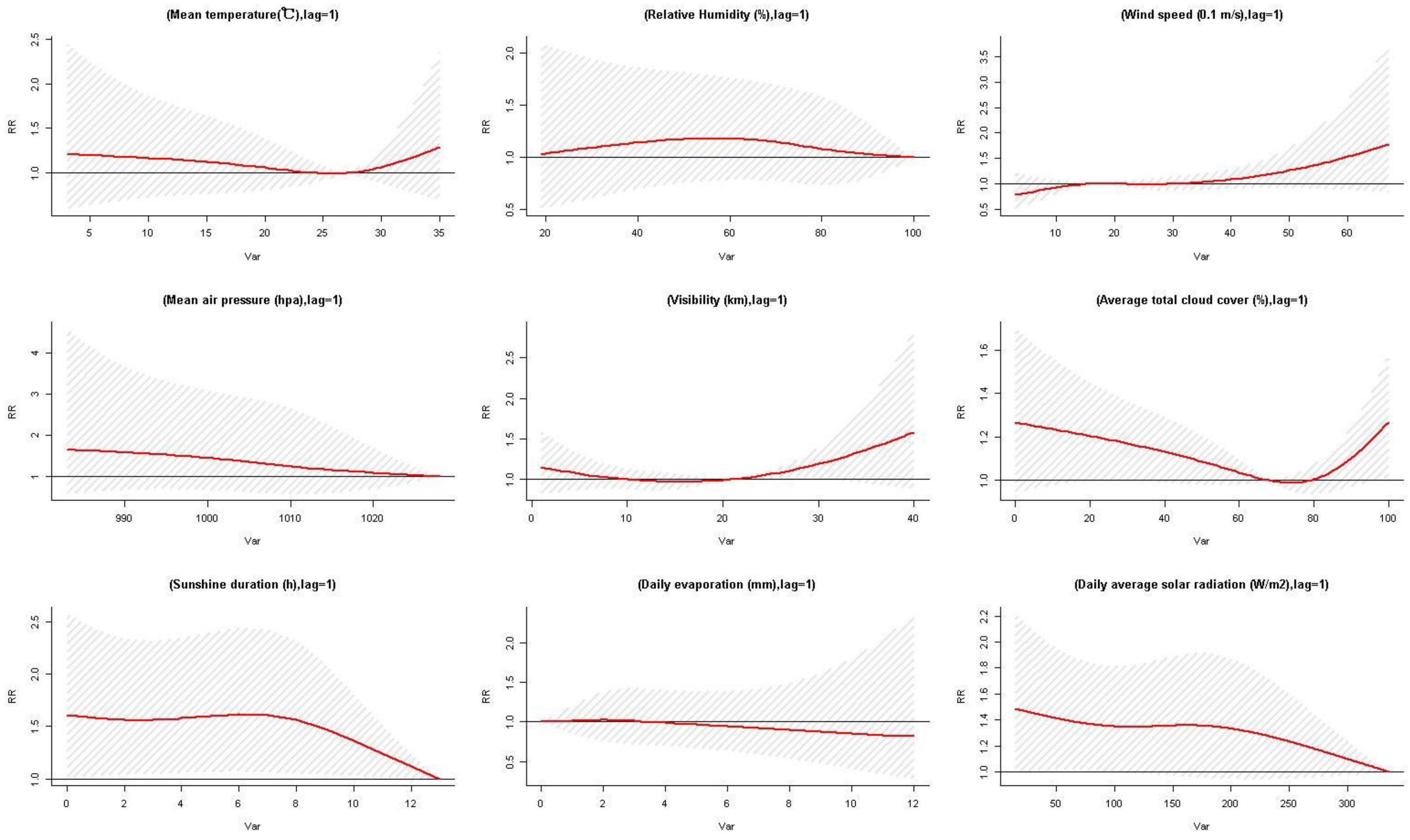

Fig.S3 Lag-response curve for lag1 effect of 9 meteorological factors on severe Bell's palsy in Shenzhen Futian district, 2009-2020

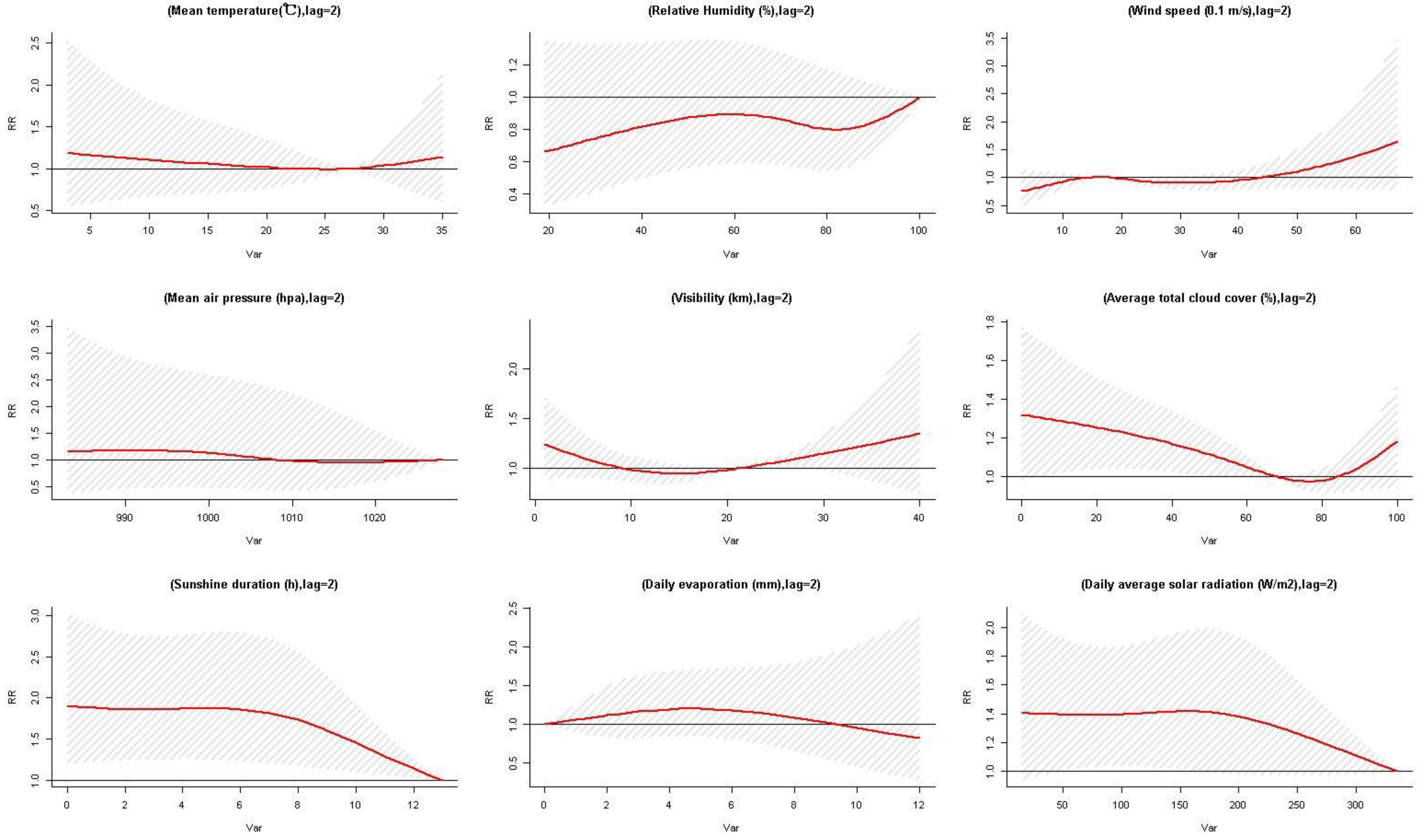

Fig.S4 Lag-response curve for lag2 effect of 9 meteorological factors on severe Bell's palsy in Shenzhen Futian district, 2009-2020

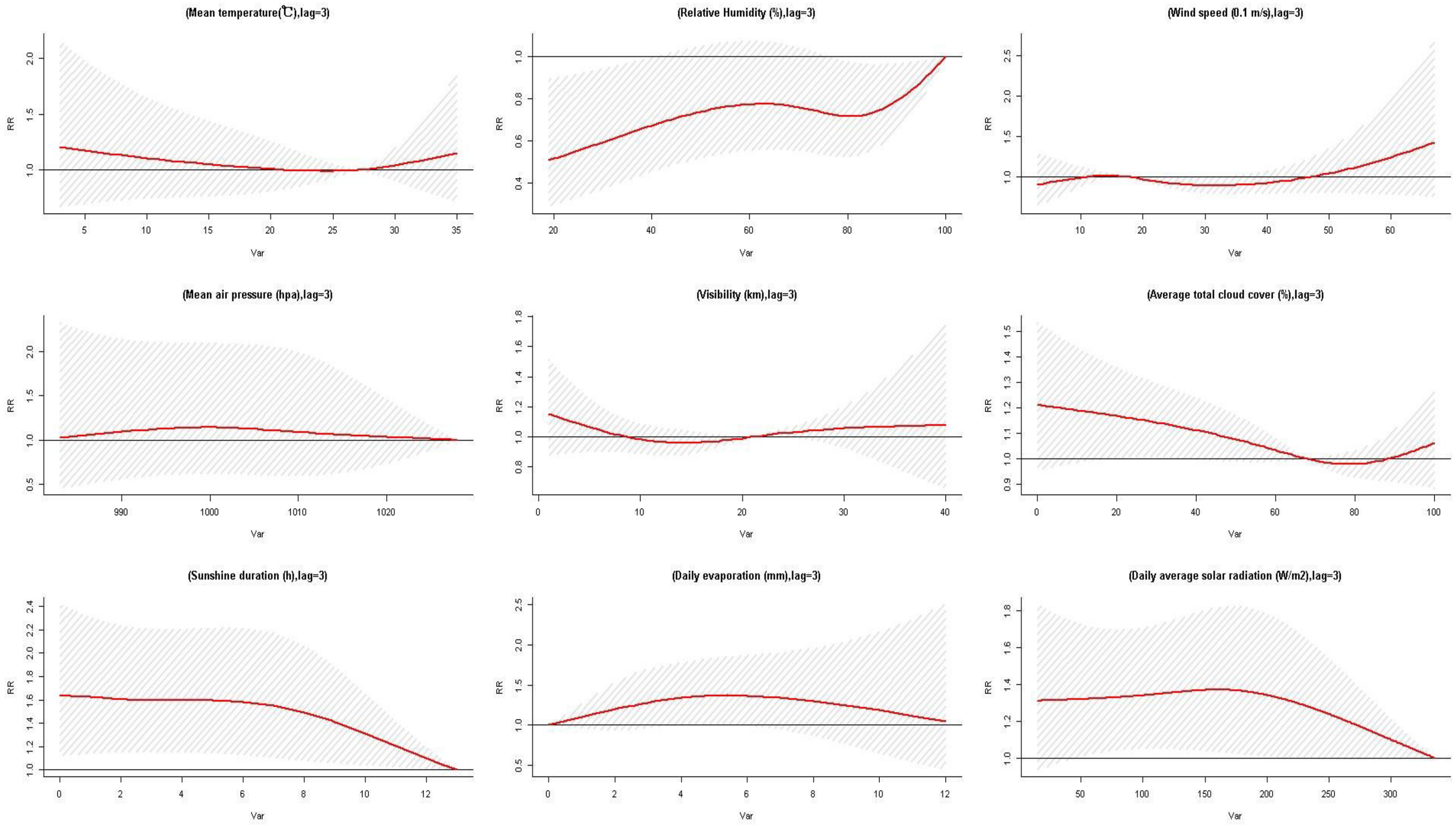

Fig.S5 Lag-response curve for lag3 effect of 9 meteorological factors on severe Bell's palsy in Shenzhen Futian district, 2009-2020

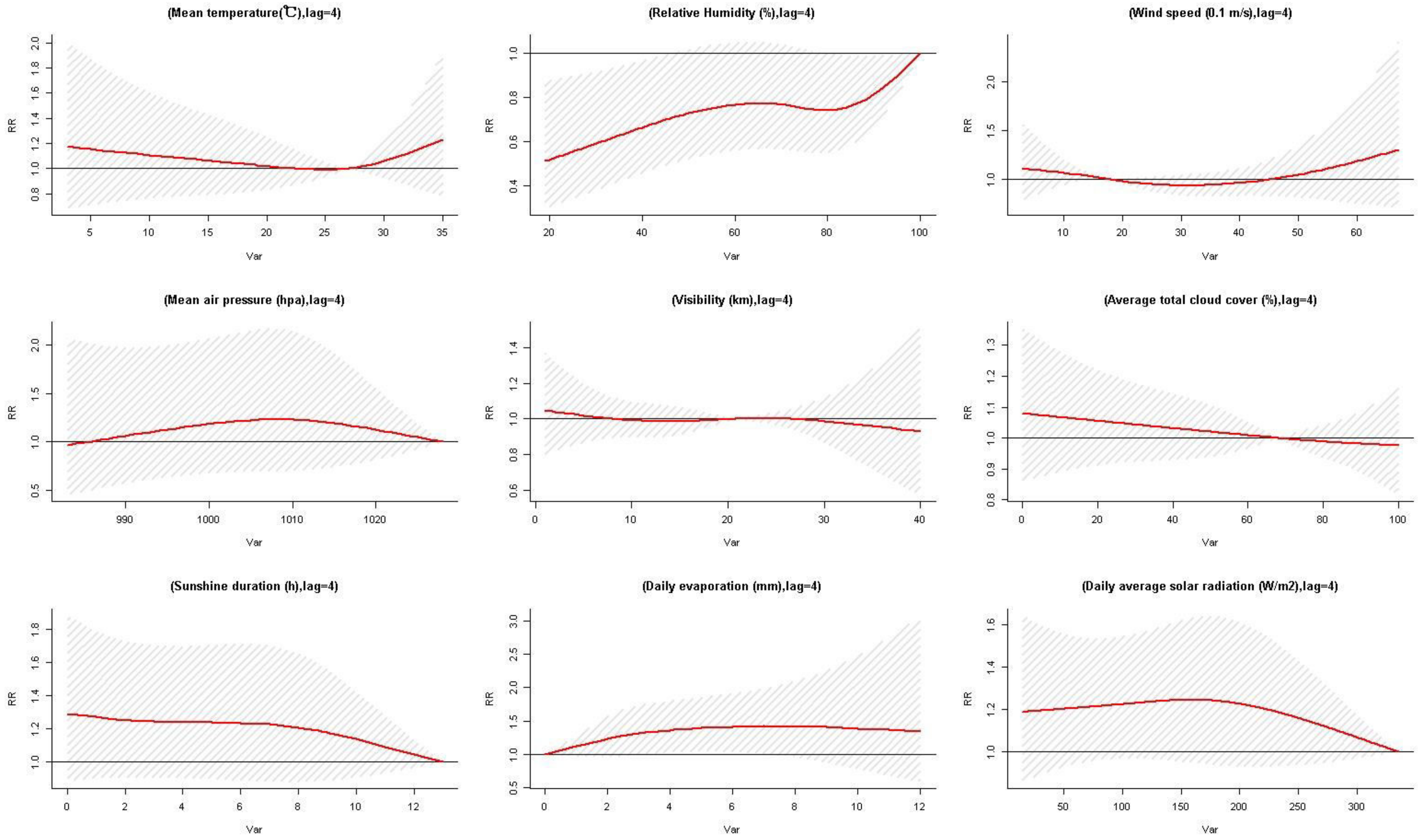

Fig.S6 Lag-response curve for lag4 effect of 9 meteorological factors on severe Bell's palsy in Shenzhen Futian district, 2009-2020

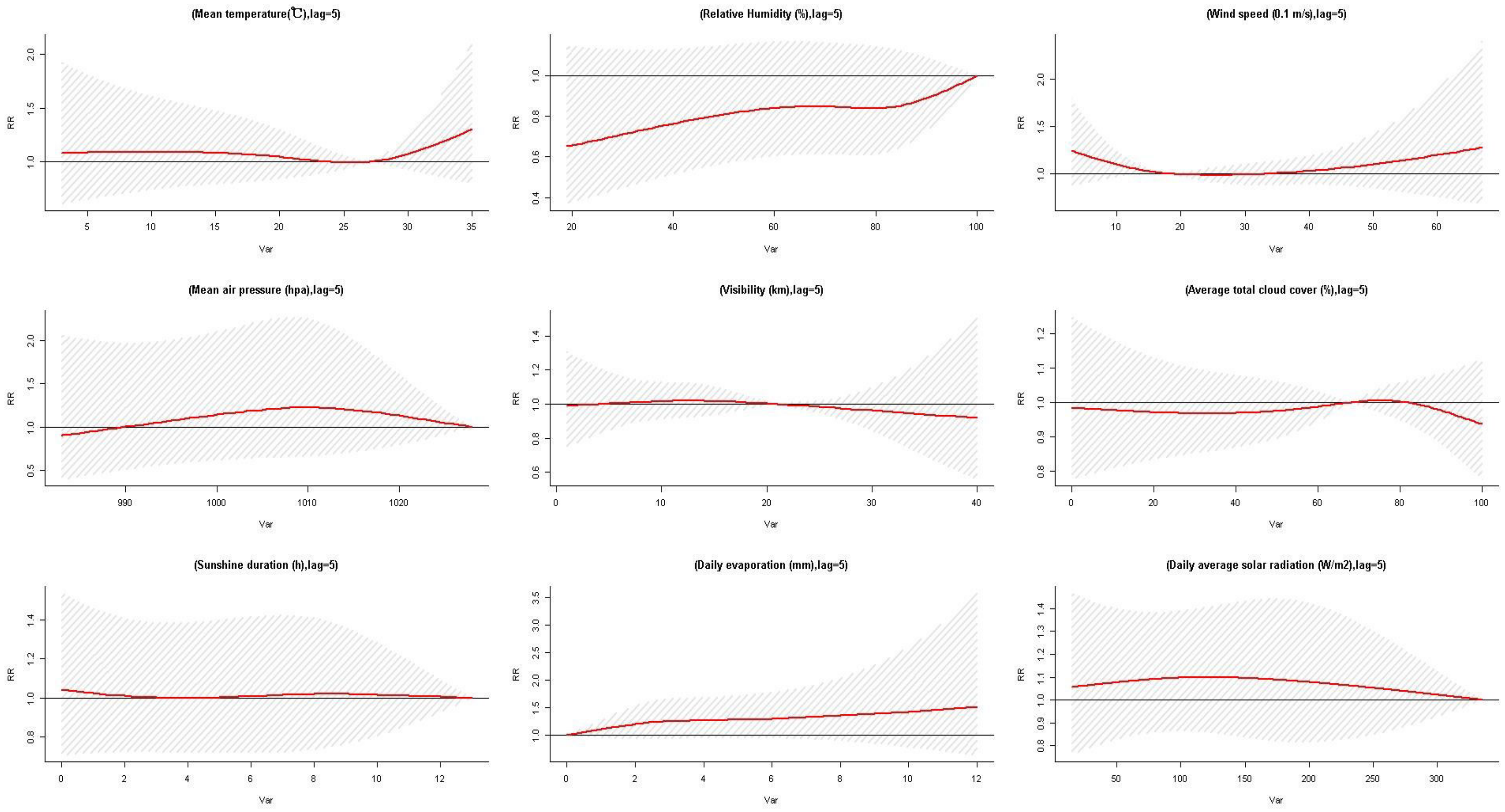

Fig.S7 Lag-response curve for lag5 effect of 9 meteorological factors on severe Bell's palsy in Shenzhen Futian district, 2009-2020

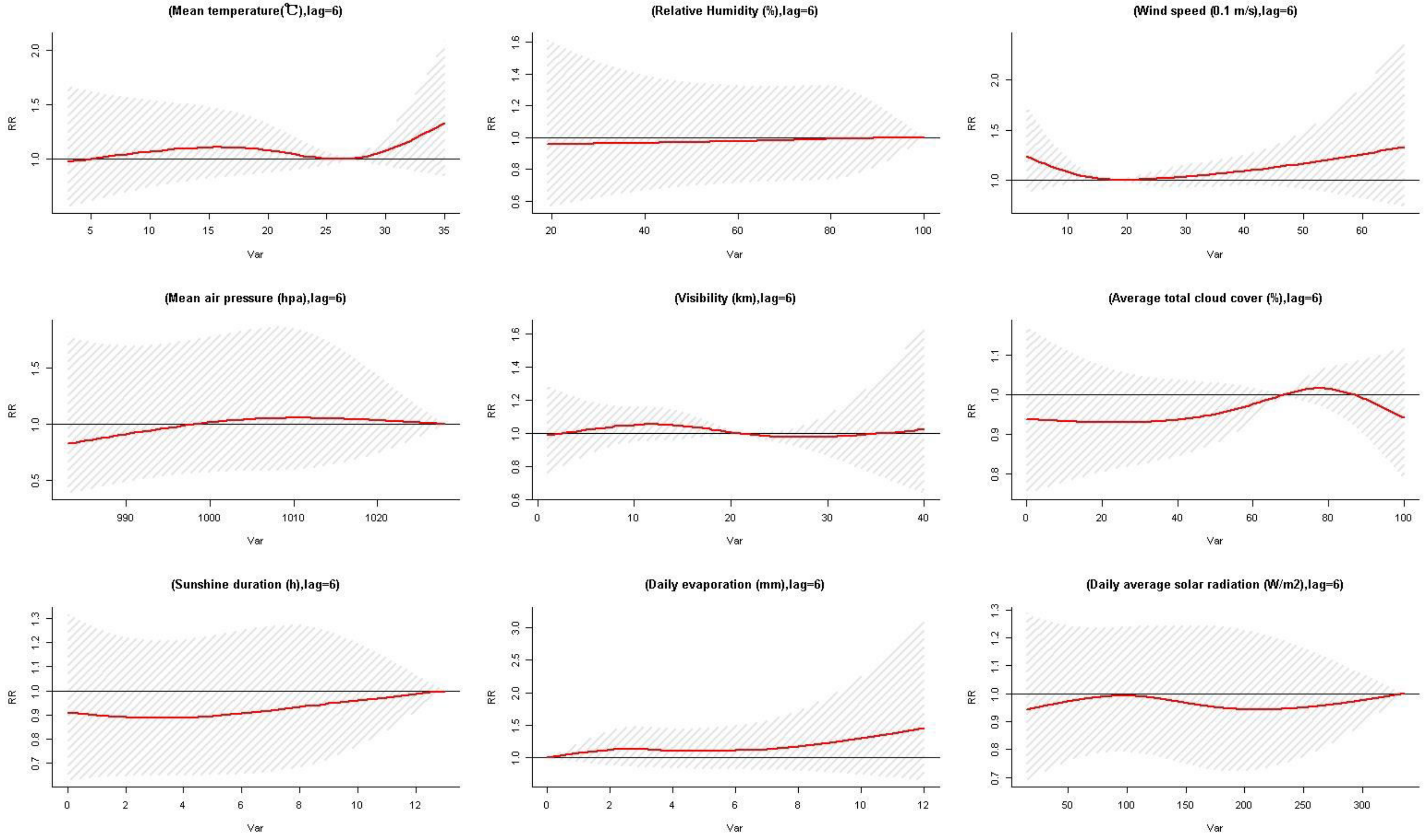

Fig.S8 Lag-response curve for lag6 effect of 9 meteorological factors on severe Bell's palsy in Shenzhen Futian district, 2009-2020

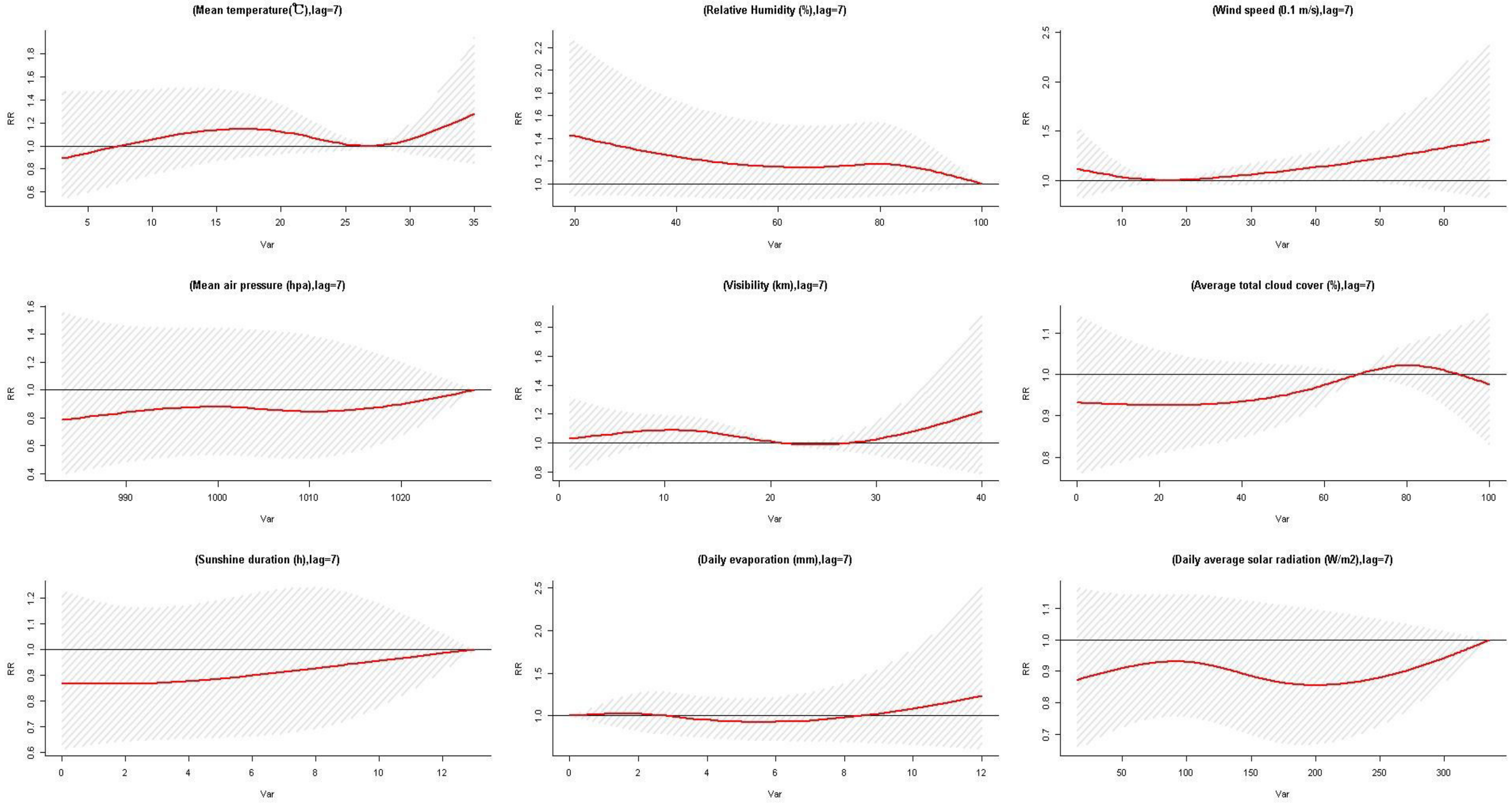

Fig.S9 Lag-response curve for lag7 effect of 9 meteorological factors on severe Bell's palsy in Shenzhen Futian district, 2009-2020

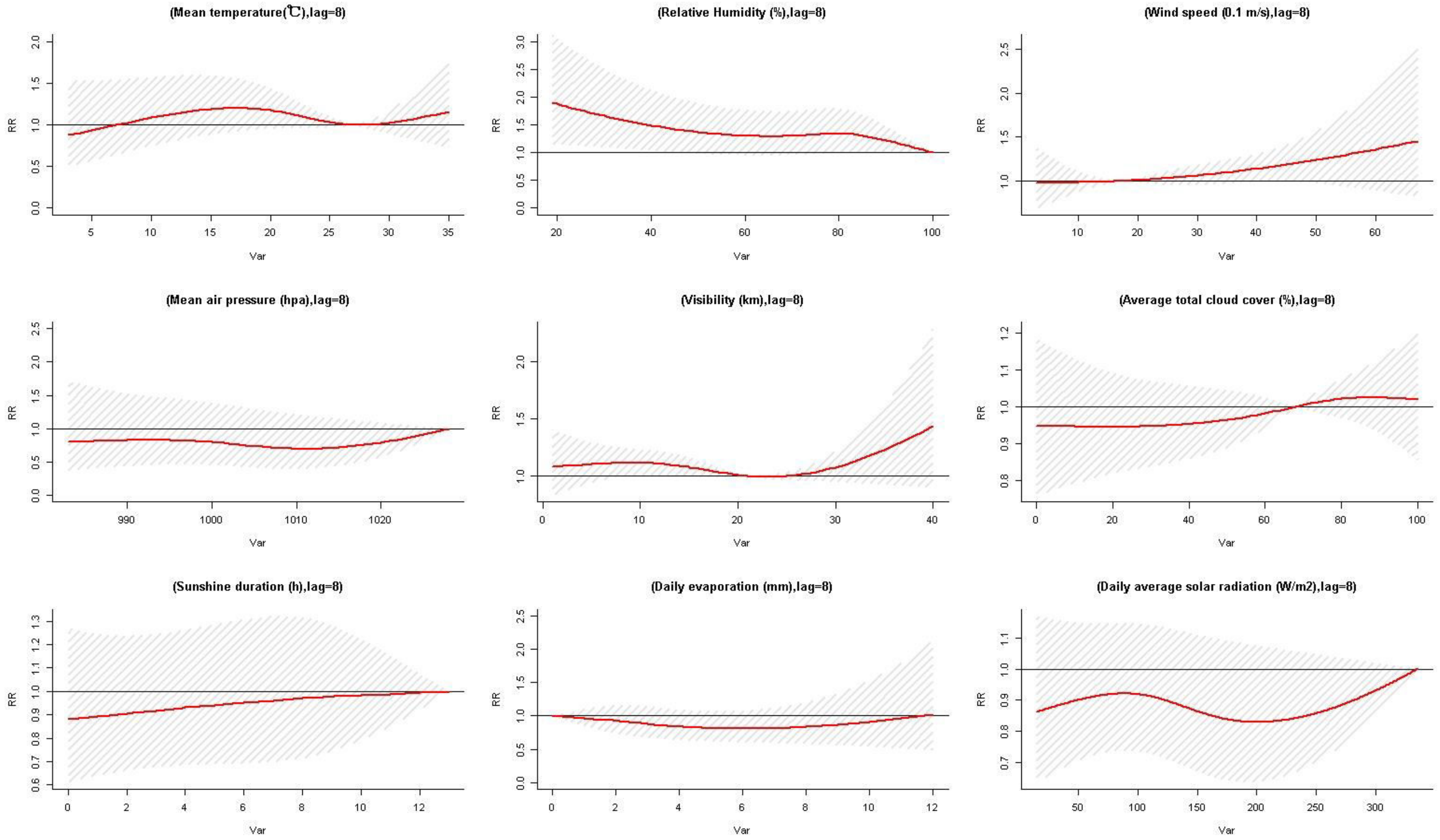

Fig.S10 Lag-response curve for lag8 effect of 9 meteorological factors on severe Bell's palsy in Shenzhen Futian district, 2009-2020

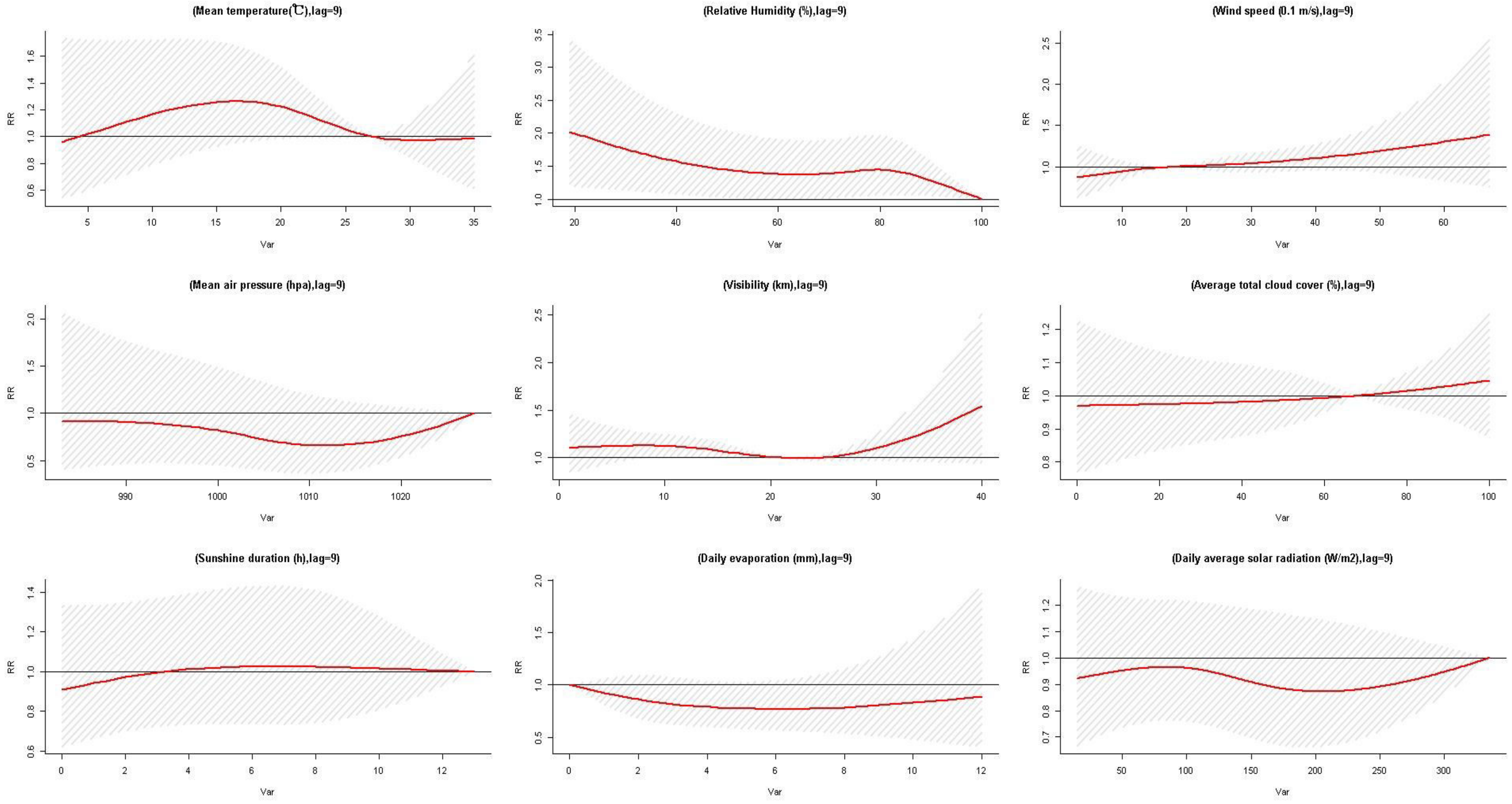

Fig.S11 Lag-response curve for lag9 effect of 9 meteorological factors on severe Bell's palsy in Shenzhen Futian district, 2009-2020

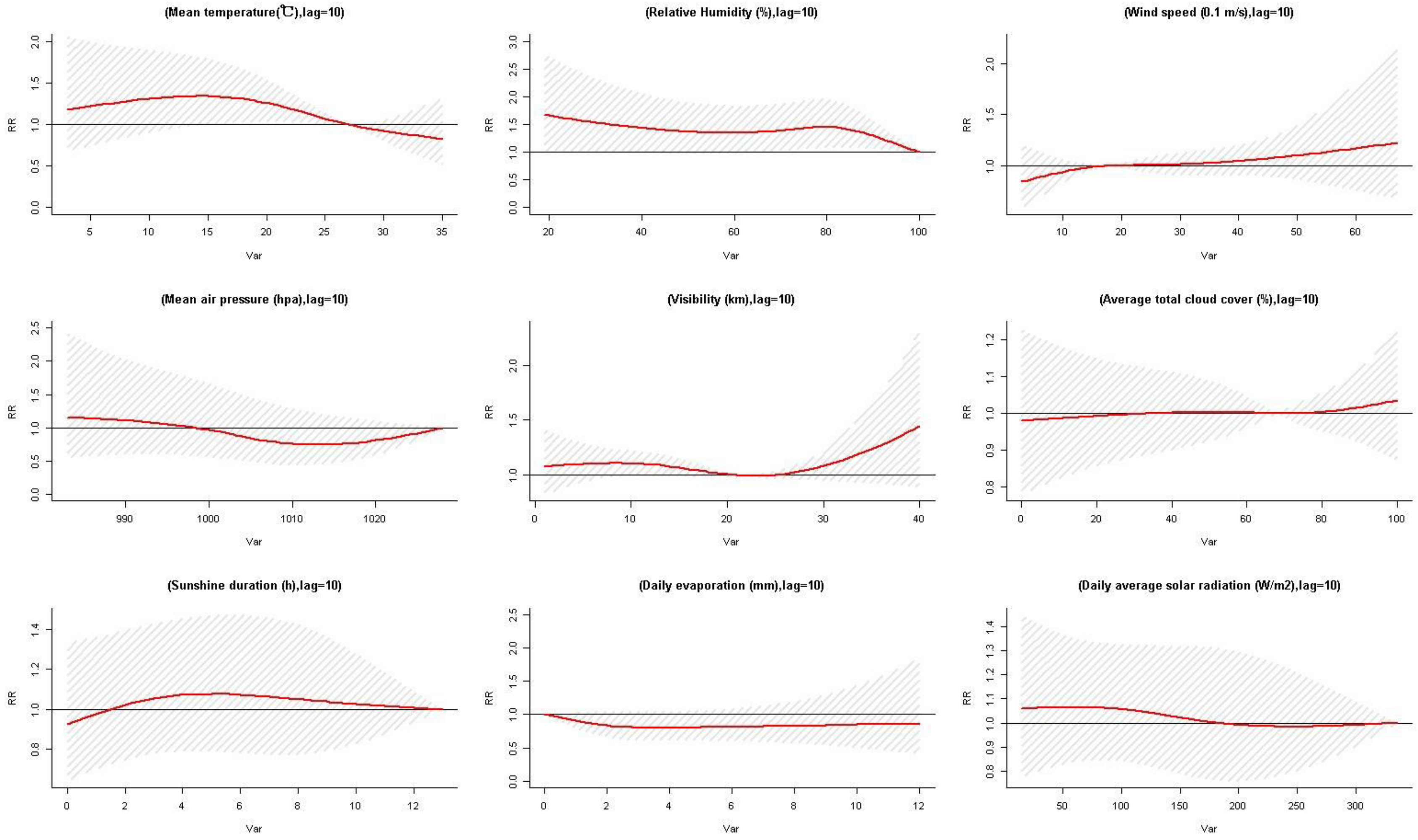

Fig.S12 Lag-response curve for lag10 effect of 9 meteorological factors on severe Bell's palsy in Shenzhen Futian district, 2009-2020

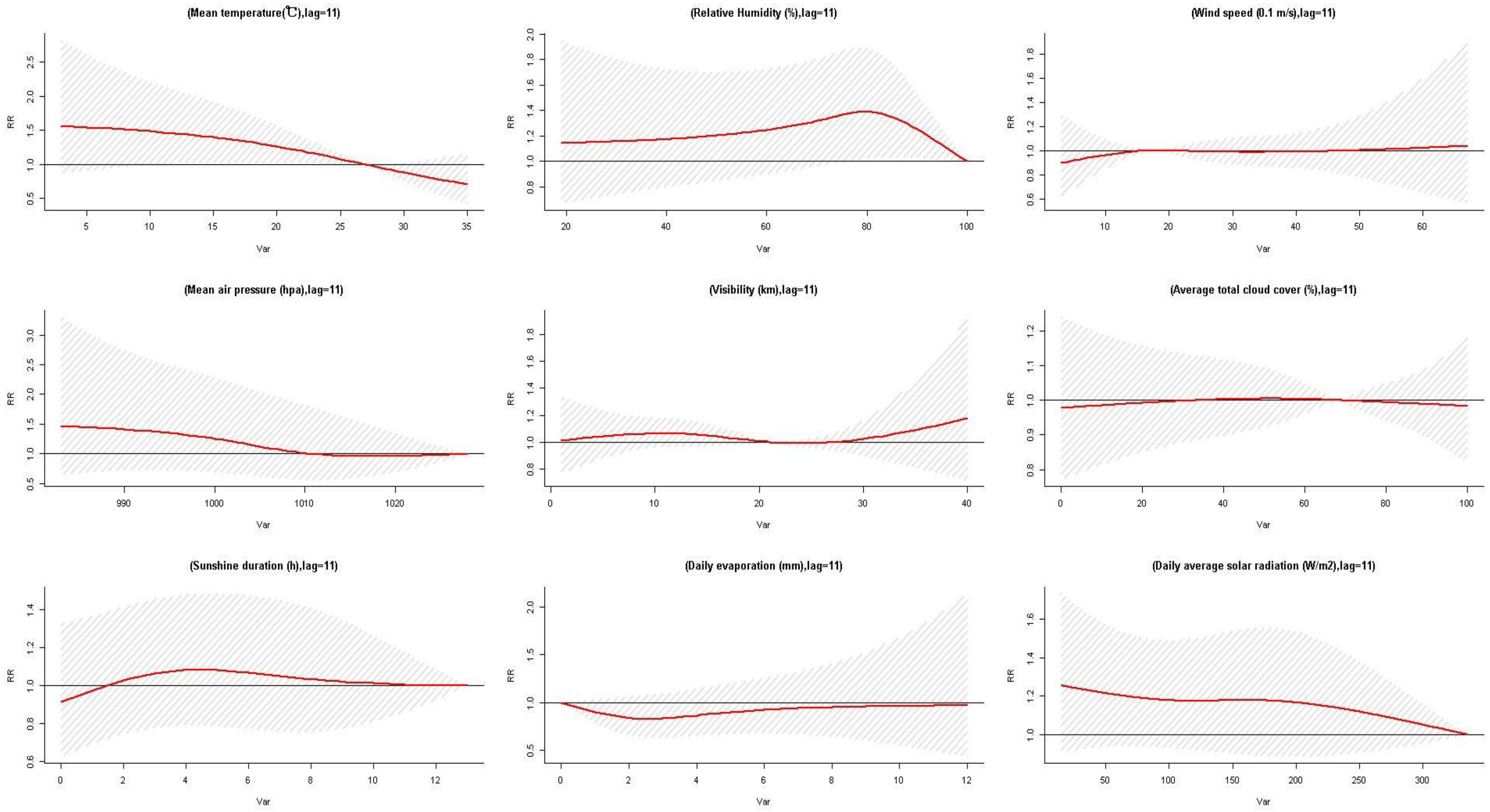

Fig.S13 Lag-response curve for lag11 effect of 9 meteorological factors on severe Bell's palsy in Shenzhen Futian district, 2009-2020

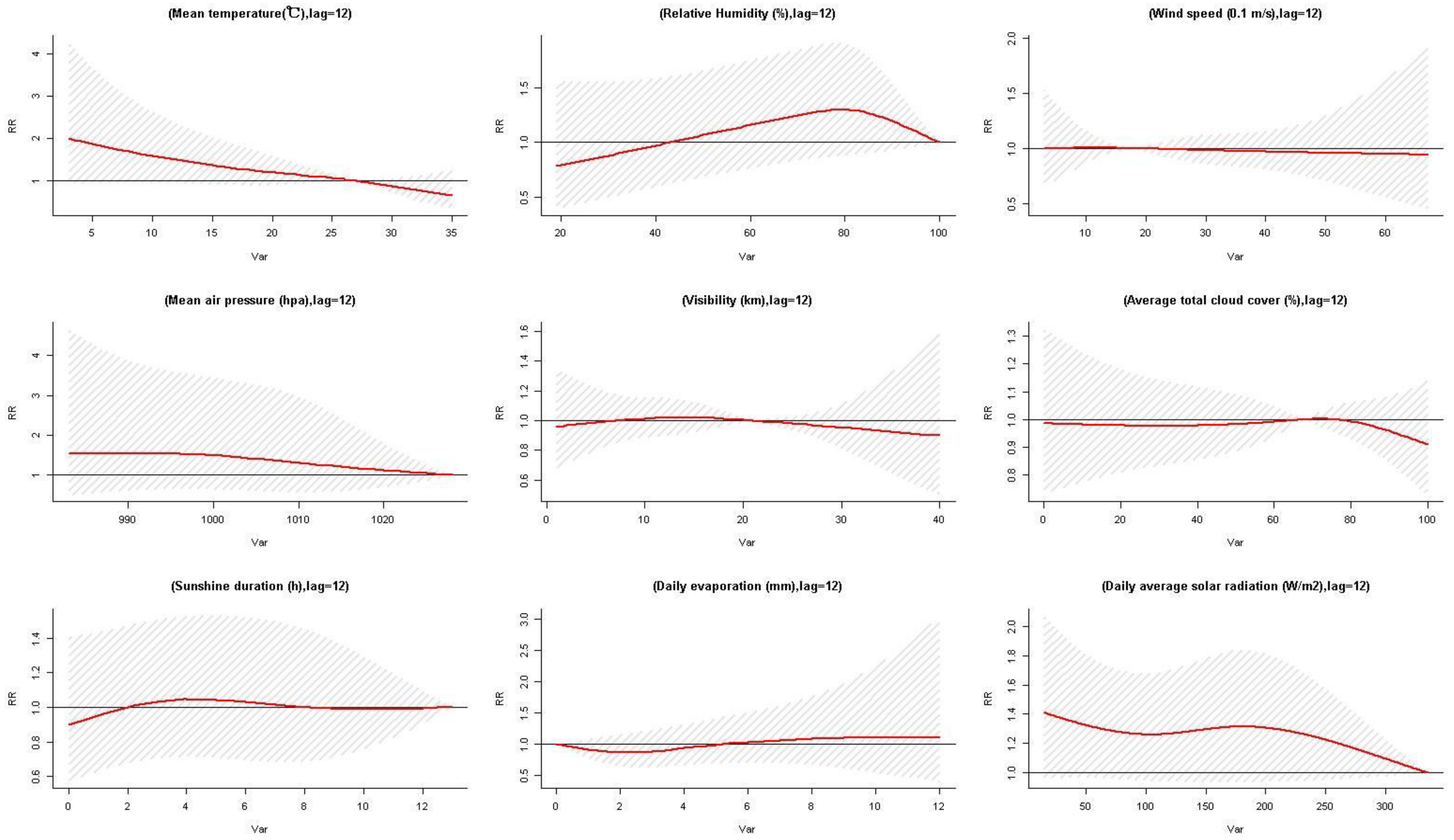

Fig.S14 Lag-response curve for lag12 effect of 9 meteorological factors on severe Bell's palsy in Shenzhen Futian district, 2009-2020

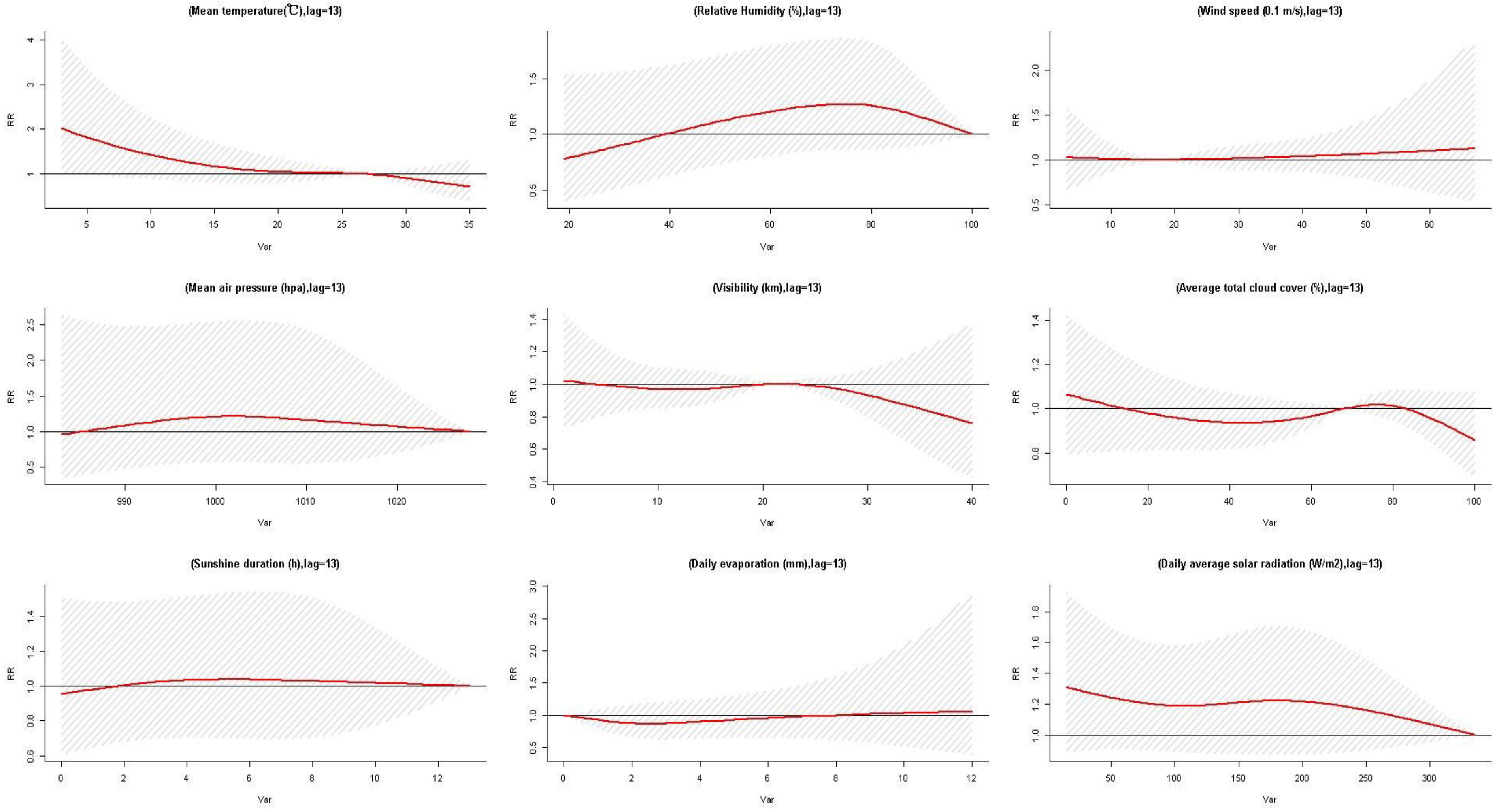

Fig.S15 Lag-response curve for lag13 effect of 9 meteorological factors on severe Bell's palsy in Shenzhen Futian district, 2009-2020

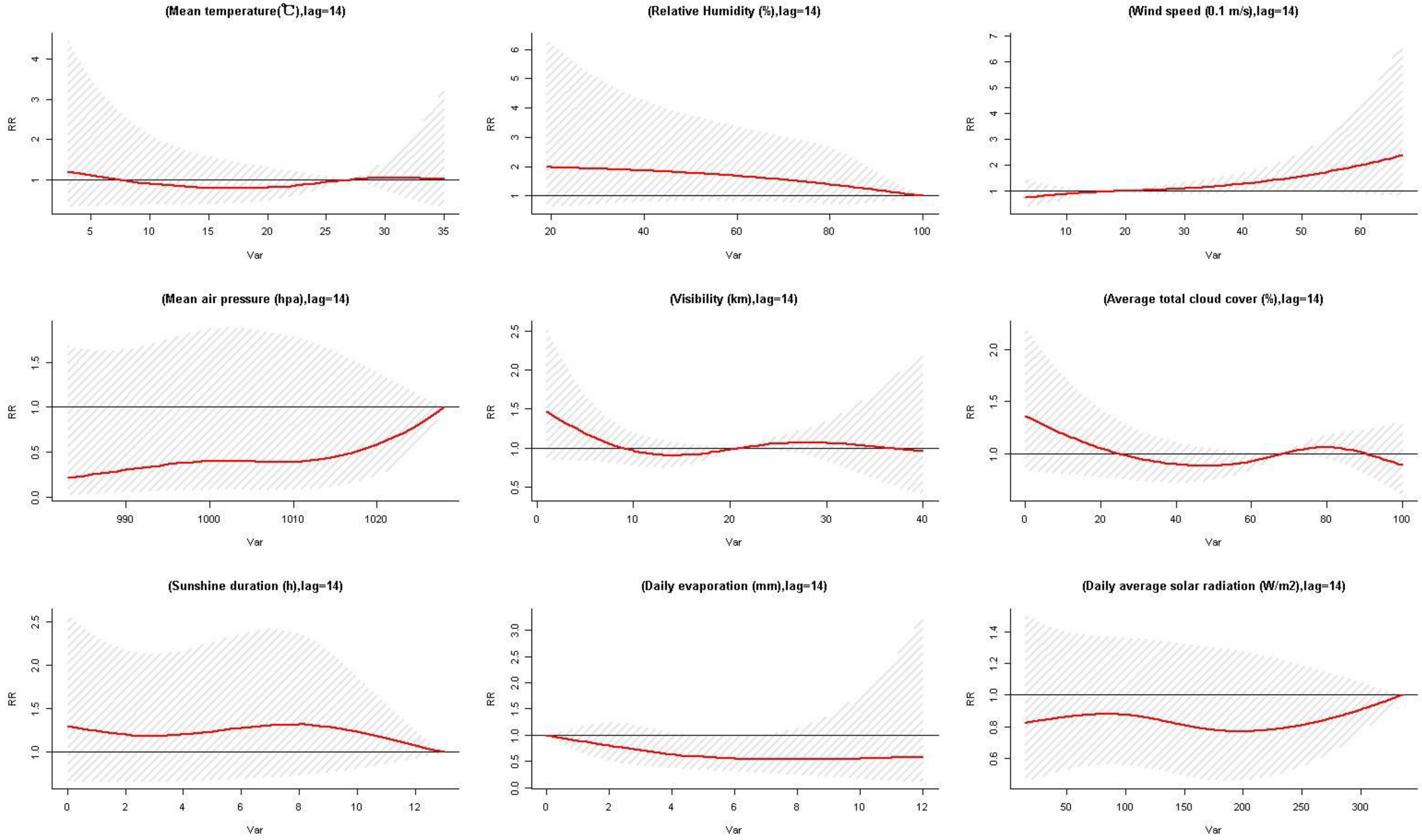

Fig.S16 Lag-response curve for lag14 effect of 9 meteorological factors on severe Bell's palsy in Shenzhen Futian district, 2009-2020
